# Survey of Motion Tracking Methods Based on Inertial Sensors: A Focus on Upper Limb Human Motion

**DOI:** 10.3390/s17061257

**Published:** 2017-06-01

**Authors:** Alessandro Filippeschi, Norbert Schmitz, Markus Miezal, Gabriele Bleser, Emanuele Ruffaldi, Didier Stricker

**Affiliations:** 1 TeCIP Institute, Scuola Superiore Sant’Anna, 56127 Pisa, Italy; emanuele.ruffaldi@santannapisa.it; 2 German Research Center for Artificial Intelligence, 67663 Kaiserslautern, Germany; nagilode@gmail.com (N.S.); gabriele.bleser@dfki.de (G.B.); Didier.Stricker@dfki.de (D.S.); 3 Junior research group wearHEALTH, Department of Computer Science, University of Kaiserslautern, 67663 Kaiserslautern, Germany; miezal@cs.uni-kl.de

**Keywords:** kinematics, sensor fusion, motion tracking, inertial measurements units

## Abstract

Motion tracking based on commercial inertial measurements units (IMUs) has been widely studied in the latter years as it is a cost-effective enabling technology for those applications in which motion tracking based on optical technologies is unsuitable. This measurement method has a high impact in human performance assessment and human-robot interaction. IMU motion tracking systems are indeed self-contained and wearable, allowing for long-lasting tracking of the user motion in situated environments. After a survey on IMU-based human tracking, five techniques for motion reconstruction were selected and compared to reconstruct a human arm motion. IMU based estimation was matched against motion tracking based on the Vicon marker-based motion tracking system considered as ground truth. Results show that all but one of the selected models perform similarly (about 35 mm average position estimation error).

## 1. Introduction

In recent years, the development of sensing technologies and sensor signals processing techniques paved the way for the use of wearable sensors to monitor human status and performance. These developments resulted in the need for managing efficiently such networks, as explained by Fortino et al. in [1]. Wearable body sensor networks (BSN) are nowadays used in several applications which include healthcare, ergonomics, sport and entertainment, (see [2] for a review on the argument). A field that has benefited from the research on BSN is motion tracking.

Motion tracking has received the attention and the effort of generations of researchers. There are several techniques that allow for motion reconstruction based on different information sources. One of the biggest challenges in motion tracking is having an accurate estimation with non-invasive sensors and non limited workspace. In the recent years, a new generation of inertial measurement units (IMUs) based on micro-electro-mechanical systems (MEMS) technology has given a new surge to motion tracking research. These devices are cost-effective and can be successfully used for accurate, non-invasive and portable motion tracking. The big interest in these devices is mainly motivated by the fact that they overcome many issues raised by optical systems and mechanical trackers. IMUs indeed do not suffer from occlusions and have theoretically unlimited workspace compared to optical motion tracking systems, and despite the accuracy of mechanical trackers, IMUs are much more affordable and far less intrusive.

Inertial units-based motion tracking has been used for navigation since decades ago. Initially developed for the attitude estimation of aerial vehicles (see [3,4]), it is nowadays used for other unmanned vehicles tracking (see [5,6,7,8]). In recent years, IMUs are often used to track human motion thus becoming an enabling technology for several applications which include localization, human-robot interaction, rehabilitation and ergonomics. This development is also witnessed by the rise of companies that sell IMUs and IMU-based systems e.g., Invensense (Invensense, San Jose, CA, USA), Trivisio (Trivisio, Trier, Germany), Microstrain (Lord Microstrain, Willistone, VT, USA) and XSens (Xsens Technologies B.V., Enschede, The Netherlands) and the amount of start-ups which target IMU-based systems. The products that they sell often include attitude reconstruction, which is provided as output to the user, or even full body motion reconstruction.

IMUs are typically composed of accelerometers and gyroscopes. These signals are used in different manners according to the applications as it will be explained in Section 2 (as an example see [9]). In most cases IMUs are used to reconstruct the pose or at least either the position or the orientation of the body they are attached to. The naive use of IMUs is the integration of the sensors’ signals over time to estimate velocity, position and orientation. Since both accelerometer and gyroscope measurements suffer from time varying biases and noises, this approach leads to a quick drift of the estimation that is unreliable after a few seconds. Therefore, researchers started investigating both algorithmic and hardware solutions to solve the drift issue. In many cases IMUs are equipped with a three axis magnetometer (e.g., [10,11,12,13]), we refer to these sensors as mIMUs. The magnetometer measures the local (earth) magnetic field that is used as an earth-fixed reference for the current estimation of the IMU orientation. Other solutions include exploiting ultrasonic sensors [14], GPS [15], ultra wide bands (UWB) [16], cameras [17], and magnetic field generated by actuated coils [18].

Motivated through the variety of approaches to IMU-based human motion tracking (IHMT), the goal of this article is introducing the reader to IHMT. In the first part (Section 2) this article introduces the reader to IHMT main issues, then it presents a survey of the methods that have been used so far to tackle the IHMT problem. In the second part (Section 3) , the article includes a tutorial section which explains in details five selected methods for upper limb tracking. This part aims at both making concrete some of the main issues presented in the survey and letting the reader familiarize with IHMT methods. These methods are finally compared to each other in Section 4. The latter section concludes the presented work.

## 2. IMU-Based Human Motion Tracking

### 2.1. Reviews on Wearable Motion Tracking

In recent decades, emerging technologies allowed for a huge step forward in human motion tracking. Exoskeletons, vision-based systems as well as motion capture based on inertial systems have become commonly used firstly in laboratory settings and nowadays in everyday life. Several reviews described human motion capture under different perspectives with a focus on the application [19] and/or on technical aspects [20]. For example, Patel in [21] proposes a review of wearable sensors for human monitoring in which a great emphasis is laid on applications and on the enabling technology. Their survey moves from sensing technology including motion capture based on inertial sensors to applications, including health monitoring, wellness and safety. Similarly, Shull et al. [22] review wearable sensing systems applied to gait analysis in clinical settings. They group methods according to the sensors that are used, subject populations and measured parameters. In a recent review paper [23], Gravina et al. discuss issues and advantages of body sensor networks, then they focus on their applications to human activity recognition. Wong et al. [24] review applications of wearable sensors to biomechanics. Differently from [21], they focus on the devices and the sensors that are used for motion tracking. Moreover, they explain advantages and disadvantages of the different methods. In the recent review [9] a specific focus is laid on wearable inertial sensors. The authors analyze several medical applications of wearable inertial motion tracking, including gait analysis, stabilometry, instrumented clinical tests, upper body mobility assessment, daily-life activity monitoring and tremor assessment. For all applications, they report the methods proposed to tackle those. Interestingly, the selection of the applications provides a grouping of methods that reflects different complexity levels in using IMU sensors: for example stabilometry requires simpler algorithms and fewer sensors than upper body mobility assessment. Harle et al. [25] provide a technical review of the issues arising and methods used in IMU-based pedestrian localization. Similarly, Yang et al. in [26] target localization, reviewing sensors as well as methods with their respective sources of errors. In [27] the focus is on walking speed estimation. Sabatini in [28] proposes a review of (m)IMU-based tracking systems for 3D attitude estimation focusing on the technical aspects of IHMT methods. In particular, sensor fusion techniques and related issues are explained in details including techniques to estimate and tune filter parameters. In [29] the authors compare six algorithms for the estimation of a smartphone’s attitude. The goal of their analysis was to compare algorithms in order to select the most suited one for pedestrian localization even when magnetometer’s signal is disturbed. They test such algorithms indoor while artificially distorting the magnetic field by means of magnets. Although authors claim that two of the selected methods perform better than the others, reported average orientation errors differ less than half of the error’s standard deviation.

The survey presented in the following subsections has a different scope and target with respect to previously published surveys: both applications and technical aspects are taken into account. Moreover, it explicitly encompasses the evolution of algorithms from the estimate of one rigid body pose to full body tracking. All reported methods are listed and characterized in the Appendix A (see Table A1). The table summarizes relevant information related to application, target, kinematic representation, sensor fusion technique and validation of each method and may help the reader in the following sections.

### 2.2. Introductory Concepts to IHMT Methods

In the late 1990s, technological advancements made inertial systems a candidate alternative to optical ones for online human motion capture. Moving from the findings in aerial vehicles navigation and accelerometry techniques, researchers started tackling the problem of human motion tracking based on (m)IMUs [30,31]. In this, major issues are: How to represent and constrain human limbs kinematics, how to fuse measurements from multiple sensors to track these with minimal drift, also considering erroneous measurements e.g., due to magnetic disturbances, and how to make the relation between technical sensor and (anatomical) body segment frames through calibration, when several (m)IMUs are involved. Moreover, ways of and measures for assessing and comparing different methods are of major interest for evaluation purposes. In the following, these major aspects and preliminary concepts of IHMT are described in more detail in order to prepare the reader for Section 2.3, which provides the IHMT survey.

#### 2.2.1. Kinematics and Constraints

All reported techniques used for inertial body motion tracking assume that human limbs are rigid bodies. Therefore, from the point of view of kinematics IHMT reduces to determining the attitude and/or the position of these limbs. When more limbs are involved a kinematic chain can be modeled. The first multi-limb models used this kinematic chain in a second step after estimating the attitude of each limb separately (e.g., [10,32]). However, the kinematic chain can be better exploited by providing joint constraints that can be added to the sensor fusion algorithm to make the estimation more consistent with human motion.

##### Kinematics Representation

While the position of a limb in space is typically represented through a Cartesian frame, several possibilities are proposed in literature to represent its orientation. Euler angles are a common choice since they have an intuitive physical meaning, which is the case of the roll-pitch-yaw representation of a vehicle attitude (e.g., [15,33,34,35]) or the identification of the roll-pitch-yaw angles with the anatomical degrees of freedoms (DoFs) of human limbs (e.g., [36,37] ). The major drawback of such a solution (and of all the three parameters attitude representations) is the existence of singularities that may occur in certain configurations, as in the case of the gimbal lock. This is why many methods use quaternions for the estimation (e.g., [38,39,40,41,42]). Quaternions allow for a computationally efficient and singularity-free attitude representation and are often used for the attitude representation of a single body. However, besides their non-minimal rotation parametrization, they do not provide direct access to anatomical or functional angles. Factorized quaternion algorithms [43,44] decompose attitude quaternions in order to identify physically meaningful rotations (e.g., about the articulations’ axes), thus allowing for a simpler implementation of constraints. Kinematic chains are indeed often parametrized either by means of Euler angles (e.g., [45]) or using the Denavit Hartenberg (DH) [46] convention (e.g., [47,48,49]) to represent the relative joint angles.

##### Constraints

Kinematic constraints play a fundamental role in the whole estimation process, as they can prevent the relative displacement of the body segments to drift over time. Kinematic constraints are sometimes embedded in the sensor fusion algorithm to provide more consistent solutions (e.g., [45,47,50,51]). In other cases the constraints are applied after the sensor fusion algorithm has provided the attitude estimation (e.g., [39]). Since the estimated quantities are often random variables, applying limits to those variables in a consistent way is a delicate issue. This is shown by Simon in [52], where different approaches to solve the issue are reviewed. In [44] quaternions are used to represent the attitudes of the human arm limbs. Anatomical constraints such as joint angle limits and limitations of the limb motions are implemented by posing the attitude estimation as an optimization problem in which the estimated attitudes have to respect the constraints and at the same time optimize the consistency with the accelerometer measurements. Also in [45,47,48,49,50,53,54], the elbow is constrained to reduced DoFs.

In contrast to the kinematic chain model, free segments models have been proposed in [51,55]. These representations keep some of the anatomical constraints as hard constraints, e.g., the connectivity between successive limbs [55], while others are relaxed (implemented as soft constraints) in order to reduce the effects of errors related to their implementation. For example, the elbow is not a perfect hinge joint as its axis is not fixed when the ulna moves with respect to the humerus [56]. Moreover, localizing the elbow axes is a further source of error [48]. On the one hand, detrimental effects of such model errors may be mitigated by the free segments approach, on the other hand this may lead to unwanted behaviours, such as measuring elbow abduction, which is not physically plausible.

#### 2.2.2. Sensor Fusion Technique

Signals gathered from accelerometers, gyroscopes, magnetometers and other sensors need suitable sensor fusion techniques to derive useful information about the attitudes and poses of the limbs. Note, sensor-to-limb calibration parameters are here assumed known. Calibration methods are addressed below. In most of the sensor fusion methods the unknown variables (e.g., Euler angles) are estimated in discrete settings at successive time steps based on previous time step estimation and current time step measures. Two main approaches are adopted for sensor fusion: complementary filters (CF) and Kalman filters (KF). More complex approaches include particle filters (PF) and optimization-based approaches, which are now suitable for online IHMT.

Complementary filters exploit the different frequency spectra of gyroscope, accelerometer and magnetometer signals. Many of the methods that exploit the CF approach (e.g., [10,30,57,58,59,60]) apply the following steps: The accelerometer signal is used to estimate the acceleration due to gravity in the sensor frame. This and the magnetometer signal are then used to obtain a “low frequency” estimate of the sensor’s attitude. At the same time an estimate of this attitude is dynamically calculated from the gyroscope measurement. These two estimates are then fused in the complementary filter. Some methods assume the body acceleration being negligible, thus modeling the accelerometer signal as a noisy measurement of acceleration due to gravity (e.g., [30,60]). In other methods body acceleration is calculated and removed from the accelerometer signal (e.g., [58]). Acceleration due to gravity and measured local earth magnetic field are then used to estimate the pose of the sensor in an earth-fixed frame (world frame). Some methods (e.g., [10]) simply implement the TRIAD algorithm [61] or TRIAD-like algorithms to reconstruct the attitude with respect to the world frame. Others [30,59,60] use more complex optimization algorithms to find the attitude as best fit of the different measurements.

The most widespread sensor fusion techniques are the Kalman Filters [62]. There are many cases in which a linear KF suffices for sensor fusion (e.g., [32,63,64,65]). In most of the cases, nonlinear equations require a manipulation of the KF. The Extended Kalman Filter (EKF) is the most immediate solution that has been adopted to use the KF approach with nonlinearities (e.g., [38,40,66,67,68,69]. Alternatively, Unscented Kalman Filters (UKF) are used in [47,49,53] as they provide a more accurate estimation of probability density functions (PDF) under nonlinear transformations. The method proposed in [70] uses unscented transformations, but implements constraints through probabilistic graphical models. Particle filters have been used by [39,42] to further improve and generalize the representation of PDFs.

Compared to the EKF, the UKF improves the estimation of the transformed probability density function. Moreover, the UKF keeps a convenient complexity when compared to the PF. Conversely, the PF allows to drop the hypothesis of Gaussian distributed random variables, thus permitting a more accurate PDF estimation. Comparisons between EKF and UKF provided conflicting results [71,72,73]. The most recent work highlighted the performance being highly influenced by the application. They describe the UKF as being more robust to initialization issues whereas the EKF is more computationally efficient [74]. The few comparisons that were found between the PF and the UKF are far from IMU-based motion tracking applications and are not reported.

Recent improvements of computational powers made optimization approaches attractive to IHMT. The methods presented in [44,51,55] show optimization based approaches that allow for both sensor fusion and implementation of constraints. Optimization approaches make it easier to take into account large time windows for the estimate. However, a good compromise has to be found between accuracy and speed of the algorithm [51] to allow for online IHMT. These latter methods are really promising because they showed to be highly flexible to add, remove, loosen or strengthen constraints as well as to find a compromise between accuracy and computational burden.

#### 2.2.3. IHMT Common Issues

There are three main issues that recur in IHMT: how to reduce the estimate’s drift, how to handle magnetic disturbances and calibration issues.

##### Drift

A very first approach to IHMT was based on inertial navigation systems (INS) strapdown integration of gyroscope measurements which was inherited from the navigation of aerial vehicles. Though adapted to follow the dynamics of a human, this solution cannot be used alone as the estimate quickly drifts. Many methods have their main focus in reducing drifts. One solution is fusing the INS or INS-like estimate with a quasi-static one, as it is done in many CF-based approaches (e.g., [58,75], see Section 2.2.2). Since drift is mainly due to gyroscope bias, a second solution is to include the bias in the estimation and to account for it [50,75,76,77,78]. A third solution exploits constraints from the kinematic chain to avoid a drifting attitude estimate of one limb with respect to the others [41,45,47,49,50,53,58,66,67,77,79]. A further solution is used mainly in lower limbs tracking and exploits contacts of the feet with the ground [38,69,78,80]. When the foot is in contact with the ground its velocity is almost null. This information can be used to reset the speed (zero velocity update, ZUPT) and, when moving on a flat ground, to also reset the height of the foot with respect to the ground. These techniques have highly reduced drifts as demonstrated in many of the aforementioned methods.

##### Magnetic Disturbances

Many of the aforementioned methods rely on magnetometers. Despite being a valuable aid to have an absolute orientation reference, their signals are easily distorted by the presence of ferromagnetic materials in the vicinity of the sensor. Distortion effects are typically classified as hard and soft iron interferences (e.g., [38,81]), which are related respectively to permanently magnetized objects and to objects that are magnetized only when an external field is applied. Hard iron effects cause an offset of the earth magnetic field whereas soft iron effects cause a distortion. If the magnetic environment does not change, these effects can be corrected through internal sensor calibration, which is out of the scope of this article. However, for dealing with a changing magnetic field, either through a changing environment or translational motion of the sensor in an environment with inhomogeneous magnetic field, several solutions have been proposed. The simplest solution is to establish a policy to decide when the magnetometer signal is reliable. This can be done by thresholding its magnitude (e.g., [82,83]). Another common solution is limiting the contribution of the magnetometer measurement to the heading variable (e.g., [45]) or to two components (e.g., [59]). A more sophisticated solution is model-based estimation of the disturbance; e.g., in [84] the magnetic field direction is estimated simultaneously with the sensor orientation. Another approach is proposed in [85]. Under the assumption that magnetic field is constant for a given period, the authors take the magnetometer measurement at the beginning of the period as a reference. They then use the error with respect to this reference at each time step to update the error state estimate in their Kalman Filter. A survey of methods to handle earth’s magnetic field disturbances is proposed by Ligorio et Sabatini in [86].

##### Calibration

All IMU based motion reconstruction algorithms require some parameters to be provided.

A subset of these parameters defines the orientations (and sometimes the positions) of the IMU frames with respect to the tracked body segments they are attached to. In most of the cases these parameters are assumed to be known: the IMU frame is supposed to be physically aligned to the body frame (e.g., [50,60,87]). In other cases these parameters are obtained by means of a calibration procedure that is carried out at the beginning of the capturing session (e.g., [47]). Another subset is related to the dimensions of the human body: human limb lengths are typically either measured (e.g., [87]), taken from anthropometric models (e.g., [64]) or calculated by means of calibration procedures (e.g., [64,88]). In contrast to the IMU-to-segment orientations and positions, there is no need for online estimation of human limb lengths as they can safely be assumed constant during tracking.

Several calibration procedures were proposed to obtain IMU and limb parameters when tracking humans. The most typical procedure requires the human to rest in the neutrum-pose (N-pose) that is standing still while leaving the arms vertical alongside the trunk in the sagittal plane (e.g., [39,45,47,89]). Another widespread calibration pose is the T-pose, where the user is standing still keeping the arms horizontal in the sagittal plane [47,64,90,91]. In [45] the user is asked to lean forward to define an earth-fixed reference frame. In [14] the user is required to assume a rest pose before each motion. Besides the static poses, functional calibration methods require the user to perform rotations around different joint axes in order to better align the body segment frames with anatomical axes (e.g., [54,92,93]).

In a clinical setting, it is particularly important to obtain anatomically interpretable joint angles and, hence, to obtain accurate IMU-to-segment orientations. In [48,94] calibration procedures comprising IMU placement protocols, static poses and functional movements are proposed for identifying the knee and elbow flexion/extension axes and the forearm pronation/supination axes, thus improving the estimation of the anatomical joint angles. A simpler calibration procedure based on two static poses (standing and sitting or lying) is proposed and evaluated in [89]. Picerno et al. [37] proposes a specific rig equipped with an IMU for IMU-to-segment orientation calibration based on anatomical landmarks. For this, the rig endpoints have to be manually placed on anatomical landmarks, which is applied to the thigh and the shank.

The effects of errors in the different calibration parameters on the limb orientation estimation errors have been recently investigated in [51] demonstrating a clear dominant role of the sensor-to-segment orientations compared to the positions and limb lengths.

#### 2.2.4. Methods’ Assessment

The assessment of motion tracking methods often relies on ground truth data, where the estimated trajectories are compared to ground truth trajectories using typical metrics, such as the root mean square error, namely RMSE, (e.g., [30,36,50,84,88,95,96,97]) or correlation coefficients (e.g., [49,53,58,87,88,96,98]). Two performance measures are mainly used for the evaluation: the drift and the accuracy of the target reconstruction, either based on the position of a reference point or the orientation of a rigid body.

Drift assessment requires to perform relatively long trials. In [36] the proposed algorithm is evaluated with respect to its drift dependency over time. In [87] the drift of the wrist position estimate is calculated and reported for one circular and one square trajectory. Luinge and Veltink [95] report attitude estimation drifts obtained from a strapdown integration of the measured angular velocities as compared to a sensor fusion algorithm using a KF. In many papers validation trials are longer than a few seconds (e.g., [10,31,38,75,92]) so that it is possible to at least qualitatively assess drift by visual inspection of the RMSE along time. Other works (e.g., [10,32,80]) do not report long assessment trials, making it difficult to evaluate drift.

Accuracy assessment requires ground truth data being at least as accurate as the method is expected to be. Single body attitude/position estimation can exploit very reliable ground truth data, such as those gathered from tilt tables (e.g., [30,43,97]). Since the IMU can be aligned very accurately to these devices, the error introduced through the evaluation device is limited and often much smaller than the estimation error. In many applications related to human motion tracking, the positions of anatomical landmarks are of interest. For this, marker-based optical motion capture (OMC) has become the gold standard (e.g., [39,40,47,48,50,55,66,77,79,91,95,99]). OMC permits to evaluate both attitude and position tracking. In the first case the main source of error resides in the alignment of the coordinate frame calculated from the OMC data with respect to the model estimation frame. The second case requires to estimate the parameters (e.g., the segment lengths) needed to calculate the reference point positions from the IMU data. Since it is not possible to measure these parameters exactly, they represent an additional source of error.

Other types of reference data were also used. One example is the work of Zhu et al. [32], in which the authors constrain the hand to follow a straight line and then check the reconstructed trajectory to be straight. Other mechanical platforms and robots were used in [42,57,75,76,81] as ground truth data.

As an alternative to tilt tables, Picerno et al. used in [100] a method for assessing the orientation estimation accuracy by attaching mIMUs to a rigid plate that is oriented in 12 different ways. They use the RMSE of the reconstructed orientation angles with respect to the known plate’s poses as accuracy metric. Devices from Xsens have also been used as providers of ground truth data. Robert-Lachaine et al. recently published [91] a comparison of MVN [64] and optical motion tracking performance when using such systems either with proprietary kinematic models or when estimating angles derived from ISB (International Society of Biomechanics) recommendations. The MVN suite was also used by Pons et al. in [41] and Taunyazov in [90], whereas the attitude estimate provided by the MTx IMU units was used by Brigante et al. in [68] and by Lee et al. in [44] to validate their methods.

It is worth noting that evaluation results always depend on the assessment method, while a great variety of such methods is currently used. Hence, it is difficult to make a fair comparison between the results reported in different publications. The summary Table A1 takes this into account by reporting the assessment methods along with the results.

### 2.3. Survey of IHMT Methods

This section provides a survey of IHMT methods categorized by the targeted body parts. This categorization allows following the historical development of IHMT methods. Indeed, starting to work on methods for a specific target (e.g., the upper limbs) and then refining it before moving to other targets represents a pattern found for many teams of researchers. Note, the presentation of the different methods combines solutions introduced in Section 2.2.

#### 2.3.1. Generic Limb Orientation

Limbs pose tracking has been tackled by means of (m)IMUs since the end of the last century. Works of Bachmann [30] and Marin [31] pioneered the field targeting orientation tracking of human limbs and robotic links by using mIMUs for computer graphics applications. The former proposed a quaternion-based attitude tracking method that updates the attitude quaternion by means of the gyroscope measurement and corrects it based on a “low frequency” estimate from the accelerometer and magnetometer measurements. Differently from [30] the second model assumes a time decay of the limbs’ angular velocities. This assumption is suitable for human motion as humans cannot maintain an average non-zero magnitude of their limbs’ accelerations for long time periods. The same group further investigated this matter with different approaches: Marin et al. in [31] moved to using an EKF to fuse a gyroscope-based quaternion attitude estimate with the estimate obtained from the accelerometer and magnetometer signals through an optimization procedure. Yun tackles the problem of limb attitude estimation in a similar way with two variants: In [97] the authors take into account a decay of human limb acceleration, whereas in [43] they adopt a factorized quaternion approach to limit the use of the magnetometer measurements for heading estimation. Both methods replace optimization with the QUEST (QUaternion ESTimator) algorithm [4] to determine the attitude from the accelerometer and magnetometer measurements. Hol et al. propose an alternative approach to IHMT for pose estimation of one limb based on UWB [101]. They develop a sensor composed of a 6-axes IMU and a UWB transmitter, whose pose estimation is the goal of the algorithm. First UWB measurements are modeled considering transmission time as an unknown, whereas gyroscope and accelerometer models are based on the kinematics of the sensor and they include time varying biases. Finally, an EKF is set to estimate the pose of the sensor. Kok et al. in [102] extend this method. They also propose a tightly coupled approach to fuse UWB measurements and IMU measurements to obtain a set of variables which includes poses of human limbs. A novel two-steps method for calibration of UWB is first proposed to obtain positions and time offsets of UWB receivers and transmitter, as well as the parameters of an asymmetric probability distribution, that they use to model measurements of UWB. Obtained UWB measurements are then used to set an optimization problem which includes IMU measurements to estimate the poses of human limbs. In [10] the upper limb posture is estimated using a CF which fuses accelerometer and magnetometer signals based on the TRIAD algorithm to reconstruct the attitude of each limb. Two nonlinear CFs are proposed in [75,76] to fuse accelerometer, magnetometer and gyroscope measurements to obtain an attitude quaternion estimation. The authors define an orientation error and demonstrate by means of a Lyapunov stability analysis that the proposed filters enforce the defined error to converge to zero. The method of Madgwicks et al. [59] is also based on a CF and includes two variations. The former uses inertial signals, while the latter uses also magnetometer measurements. The method with magnetometers is described in Section 2.2.2. It exploits an earth-fixed frame to reconstruct the IMU’s orientation as a quaternion q. The relation of its time derivative with the angular velocity allows the authors to use the gyroscope measurements for the CF “high frequency” estimate of q. The “low frequency” estimate is obtained from an optimization procedure in which the goal is to align vectors measured in the sensor frame with their known counterparts in the Earth fixed frame. This second part can be adapted depending on the availability of measurements; e.g., acceleration due to gravity in case of an IMU and, in addition, local magnetic field in the case of an mIMU. Finally, the method of To and Mahfouz [42] tries to improve the quaternion attitude estimation by using von Mises-Fisher and Bingham densities in a PF that provides the attitude quaternion based on the IMU signals.

#### 2.3.2. Lower Limbs Tracking

IMU-based lower limbs tracking has been tackled for several purposes. In some cases it has been used for gait analysis [36,37,79,93], in other cases only parts of the lower limbs were targeted, mainly for monitoring purposes in medical settings [94,96] or rehabilitation [44,59]. In [88] a system for tracking shank and thigh orientation in the sagittal plane is presented. The authors use two IMUs (both the accelerometers and the gyroscopes were biaxial) attached to these body segments. They perform direct integration with updates according to the difference between the detected acceleration and the acceleration due to gravity.

The work in [63] aims instead at estimating the knee flexion/extension angle based on IMUs attached to the user’s thigh and shank. They use KFs to estimate the IMUs’ attitudes and model the knee as a hinge joint to obtain the flexion/extension angle assuming the orientations of the sensors with respect to the knee joint to be known. Similarly, in [98] the target is knee angle estimation, and the IMU poses with respect to the knee rotation axis are supposed to be known or determined through a calibration procedure. Favre et al. show an application of a similar approach to knee ligament injury monitoring [92,96]. The same authors further developed their method to overcome calibration issues. In [103] they propose a functional calibration procedure to obtain clinically relevant joint angles. The importance of calibration (see Section 2.2.3) for measurements in clinical settings is further witnessed by the works of Picerno et al. [37] and Cutti et al. [48,94] who developed calibration procedures to map mIMU-based 3D kinematics reconstruction to anatomical landmarks. Knee angle estimation based on two IMUs on thigh and shank is also the target of Seel et al. in [93]. They propose a calibration procedure that allows to obtain the knee joint position and knee flexion/extension axis in the sensors’ frames. Based on this, they propose two magnetometer-free joint angle estimation methods. The first method exploits IMU orientation estimation to obtain the knee angle as orientation difference about the knee axis. The second method exploits directly the hinge joint assumption to obtain the knee angle by integrating the difference of the angular speeds with respect to the knee axis. Finally, drift is removed by an acceleration-based joint angle estimation. They test their method against ground truth from an OMC system by mounting IMUs and optical markers on both human and prosthetic legs.

Lower limbs reconstruction has often been used to aid localization during locomotion. Examples include the methods presented in [65,104]. The first exploits detection of contacts and a lower limbs biomechanical model to correct acceleration and velocity errors. Localization is then obtained by integration of linear velocity. The second implements KFs too estimate limbs orientations from mIMUs signals. KFs estimate IMUs biases and the errors of limbs orientation quaternions, which are then used to correct orientation estimate from INS. The method implements also ZUPT and adaptive weighting of accelerometer and magnetometer signals to mitigate detrimental effects of linear acceleration and magnetic field disturbances. Estimates from left and right legs are finally merged by a KF to obtain pelvis displacement.

Joukov et al. propose to use five IMUs to track locomotion for gait analysis [79]. They use two kinematics models to model the support and the swing leg. The first connects the feet to the ground by means of hinge joints (stance leg), whereas the latter connects the waist to the ground by means of three prismatic and three hinge joints. IMU data are fused in an EKF whose states comprise the joint variables and their time derivative. The method is validate on ten cycles but only knee joint angles are reported.

Zihajehzadeh and Park in [105] propose a method that substitute magnetometer with UWB. They use 7 IMUs attached to feet, shanks, thighs and pelvis as well as 3 UWB tags attached to feet and pelvis to reconstruct lower limbs motion and localize pelvis. Interestingly they exploit the robustness of the estimate of limbs’ inclination to remove yaw estimation drift. Their method moves from a first KF (tilt KF) that estimates inclination of the seven limbs based on accelerometer an gyroscope signals. Then they use UWB for a second KF whose output are feet positions and yaw of feet and pelvis. This output and tilt KF output are finally used to estimate shanks and thighs yaw. They obtained good results (orientation error below 5${}^{\circ }$ and position error below 5 cm) walking, jumping and ascending validation trials.

#### 2.3.3. Upper Limbs Tracking

More than a decade ago, Luinge and Veltink in [95] exploited IMUs to track the orientation of the upper limbs by modelling the accelerometer and gyroscope measurements as a function of attitude, biases and noises and using a KF to estimate orientation errors and biases based on these models. This method was applied in [54] for tracking the relative orientation of the forearm with respect to the upper arm. Other works from these and associated researchers addressed magnetic disturbance handling [84] and extending the method to full body motion tracking [64].

Mihelj [50] used IMUs to track human arm motion in a rehabilitation task. In this task the user’s hand was firmly fixed to a robot and the known hand pose was used to complement the IMU information. mIMUs were also used by Jung et al. in [67] to track the motion of the trunk and the upper limbs. These were modeled as two four DoF serial kinematic chains which were connected to the trunk, while the latter had three rotational joints with respect to the pelvis.

Bleser et al. proposed in [45] a novel method for upper limbs tracking that exploits an egocentric camera and markers to aid mIMU-based estimation. The topic was then further investigated addressing motion tracking algorithms for general kinematic chains [66], investigation of the effects of different model calibration errors and biomechanical model representations on the segment orientation estimation accuracy [51] (studied based on arm motions), simultaneous motion and IMU-to-segment calibration estimation [106] as well as low-cost full body sensor suits [107]. Targeted applications include ergonomics in industrial manufacturing [108] and rehabilitation [109].

Peppoloni proposed in [47] an mIMU-based method for arm tracking, modeling each shoulder and elbow with five DoFs and using an UKF to fuse the mIMU data. In [70], the same group proposed a method where the UKF was replaced by a probabilistic graphical model approach. The method takes into account the constraints provided by the kinematic chain model and implements a message passing approach to estimate the joint angles. Considered applications include ergonomics [110], robot teleoperation [111] and rehabilitation.

Particle filters were used by Zhang et al. [39] to fuse inertial and magnetometer measurements for estimating the elbow flexion/extension angles. The same authors worked previously on an UKF-based method presented in [49].

The upper limbs tracking approach of El Gohary [53,77] exploits IMU measurements fused in an UKF. The method was eventually improved [78] by including IMU biases and ZUPTs to limit drift.

Taunyazov et al. [90] adopted a simpler approach to track the upper limbs. Their method relies on one IMU mounted on the upper arm and a simple mechanical tracker equipped with a potentiometer to measure the elbow’s rotation angle.

Finally, upper arm pose estimation is the goal of the methods analyzed in Section 3, i.e., [32,45,47,58,97] which are all based on mIMUs and exploit different calibration and sensor fusion techniques.

#### 2.3.4. Full Body Motion Tracking

Some of the aforementioned methods were extended to full body motion tracking. Works from professor Veltink’s group led to the development of a commercially available inertial body tracking system based on a body suit with 17 mIMUs [64] (the Xsens MVN system). The motion reconstruction algorithm also benefits from the work of Schepers, Roetenberg and Slycke on the exploitation of disturbed magnetic field signals [99,112].

Vlasic et al. [14] developed a full body suit equipped with 18 IMUs and eight ultrasonic sources. The IMUs were equipped with microphones so that the received signals provided a reference to avoid the drift that would occur when purely integrating accelerometer and gyroscope measurements.

The full body tracking method of Pons-Moll et al. [41] is mainly based on camera images. The limbs poses are inferred from the video information within a set of possible poses. This set is reduced thanks to the orientation cues from IMUs mounted on the body.

A linear CF is proposed in [58] in which the orientation quaternion of each limb is obtained as a linear combination of an estimate based on the gyroscope measurements and one based on the accelerometer and magnetometer measurements.

Miezal et al. [66] exploited an EKF to develop a general framework for motion tracking of arbitrary kinematic chains based on mIMUs.

An interesting probabilistic method has been developed by Kok et al. in collaboration with XSens [55]. Instead of using a recursive filter, joint angles are estimated from IMU measurements using constrained optimization. Here, constraints from the biomechanical model and from assumptions about the average acceleration over time are included into the cost function as both hard and soft constraints. Moreover, errors due to sensor shortcomings and soft tissue artefacts are modelled by incorporating appropriate noise terms. The maximum a posteriori estimate is obtained in an offline process using an infeasible start Gauss Newton method to solve the weighted least squares problem. Recently, Miezal et al. [51] proposed a variation of Kok’s offline method to enable online constrained optimization using a sliding window approach.

Multiple limbs and full body suits have been applied to several fields. In the sports field, for example, Ruffaldi et al. in [113] use IMUs to analyze rowing performance by estimating the rower’s motion based on five mIMUs. Measurements from the rowing simulator hardware (oars and seat) aids the overall estimate. Supej et al. developed a full body suit based on Xsens MTx sensors to track ski performance [114]. In [57] Miller et al. addresses remote robot control through IMU-based motion tracking. YostLabs (YostLabs, Portsmouth, OH, US), formerly YEI technologies, distributes full body IMU-based motion tracking applied to computer graphics and Virtual Reality (PrioVR). The system enables computer game players to control virtual characters through their own motions (see YEI technology, http://www.yeitechnology.com/).

## 3. Selected Methods

This second part of the article is in the form of a tutorial, and provides more details on five methods which have been selected in order to span the different areas that were identified in Section 2. These methods differ concerning the sensor fusion technique, using either CF, KF, EKF or UKF. They also differ concerning the sensors that are used: all of them exploit IMUs, but magnetometer signals are not always used and one method requires a visual reference for tracking human upper limbs. They also differ regarding the kinematic models: some use Euler angles, some use the DH convention and others use quaternions. Moreover, they differ in how the constraints of the kinematic chain are considered. Finally, they differ in how the parameters of the algorithms are set. In the following these methods are briefly recalled, more details can be found in the cited papers.

The following notation will be used. The *i*-th accelerometer signal will be ${\tilde{a}}^{i}$, the gyroscope’s will be ${\tilde{\omega }}^{i}$ and the magnetometer’s will be ${\tilde{m}}^{i}$. Vector a will denote linear acceleration of a point. The earth magnetic field and the gravity vectors will be respectively m and g. pl will specify that the vector p is written in the reference system *l*. $I_{n}$ will be the size *n* identity matrix, $0_{n,m}$ a *n* by *m* null matrix, and *T* is the sample time. The quaternion $q^{i}$ will represent the attitude of the *i*-th body.

### 3.1. Method 1

**Figure 1:**
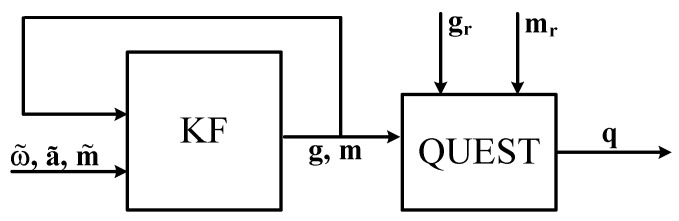
Diagram of method 1 for the *i*-th limb, the diagram represents one temporal slice of the motion reconstruction. Vectors $g_{r}$ and $m_{r}$ are the gravity and the magnetic field vectors represented in the *i*-th limb frame in a reference configuration, e.g., N pose.

The first method that is described in [32] is suitable for the reconstruction of an arbitrary kinematic chain, given that each link is provided with a nine axis mIMU. Figure 1 shows the block diagram that summarizes this method. Given two consecutive links in the kinematic chain, namely *i* and i+1, each one provided with a frame $\tau_{i}\equiv \left(O_{i},x_{i},y_{i},z_{i}\right)$, the authors represent the orientation between the two frames by a axis-angle representation with axis $k_{i}^{i+1}$ and rotation angle $\theta_{i}$, from which the rotation matrix $R_{i}^{i+1}$ can be obtained.

The rate of change of $g^{i}$ and $m^{i}$ within the same frame $\tau_{i}$ can be calculated as

(1) $$\begin{array}{cc} {}^{i}\dot{g} & =S\left(\omega^{i}\right){}^{i}g\\ {}^{i}\dot{m} & =S\left(\omega^{i}\right){}^{i}m \end{array}$$

where $\omega^{i}$ is the angular velocity of the frame $\tau_{i}$ and S(ω) is the skew-symmetric matrix of vector ω

(2) $$S\left(\omega \right)=\left[\begin{array}{ccc} 0 & -\omega_{z} & \omega_{y}\\ \omega_{z} & 0 & -\omega_{x}\\ -\omega_{y} & \omega_{x} & 0 \end{array}\right].$$

It is worth noting that the gyroscope measurement is treated here as a known control input, thus not taking into account the gyroscope measurement noise.

Under the assumption of slow motion (or that the linear acceleration is known) and that the *i*-th sensor frame is aligned with $\tau_{i}$, then $g^{i}$, $m^{i}$ and $\omega^{i}$ are approximately the output of the *i*-th sensor, i.e.,

(3) $${}^{i}g\approx {\tilde{a}}^{i}\;\mathrm{and}\;{}^{i}m\approx {\tilde{m}}^{i}$$

The authors hence propose to use a KF for each segment in which the state is

(4) $$X^{i}=\left[\begin{array}{c} {}^{i}g\\ {}^{i}m \end{array}\right].$$

The process model between steps *j* and j+1 is derived from Equation (1):

(5) $$X_{j+1}^{i}=\left({\dot{X}}_{j}^{i}T+I_{6}\right)X_{j}^{i}+w_{j}$$

where $w_{j}$ is white noise. According to Equation (3) the measurement model is hence

(6) $$Z_{j}^{i}=X_{j}^{i}+\delta_{j}^{i}$$

where and $\delta^{i}$ is the white measurement noise.

The KF estimation of ${}^{i}g$ and ${}^{i}m$ feeds the QUEST algorithm to calculate the attitude quaternion $q^{i}$. The resulting quaternion $q^{i}$ is converted to a rotation matrix that feeds the homogeneous matrices

(7) $$A_{i}^{i+1}=\left[\begin{array}{cc} R_{i}^{i+1} & {}^{i}d_{i}^{i+1}\\ 0 & 1 \end{array}\right],$$

in which ${}^{i}d_{i}$ is the position of $O_{i+1}$ in the frame $\tau_{i}$, are then recursively applied from the chain root up to the desired point to obtain its position in the root frame.

### 3.2. Method 2

**Figure 2:**
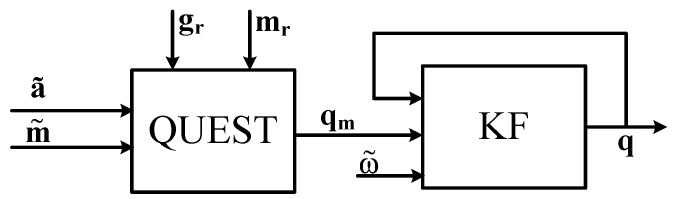
Diagram of method 2 for the *i*-th limb, the diagram represents one temporal slice of the motion reconstruction. Vectors $g_{r}$ and $m_{r}$ are the gravity and the magnetic field vectors represented in the *i*-th limb frame in a reference configuration, e.g., N pose.

In the method proposed by Yun et al. [97] each limb is supposed to be independent of the others and equipped with a nine axes mIMU sensor. Figure 2 shows the block diagram that summarizes this method. The attitude of *i*-th limb with respect to the root frame is represented by a quaternion $q^{i}$. Under the assumption that the linear acceleration of the human limbs is negligible with respect to the gravity, and that the mIMU axes are aligned with the limb ones, then $q^{i}$ is initially estimated by means of the QUEST algorithm fed by equally weighted accelerometer and magnetometer signals thus obtaining $q_{m}^{i}$. To compensate for the dynamic effect of the linear acceleration, the authors estimate the rate of change of $q^{i}$ based on the link angular velocity $\omega^{i}$ also measured by the mIMU:

(8) $${\dot{q}}^{i}=\frac{1}{2}q^{i}⊗\omega^{i}=Q\left(\omega^{i}\right)q^{i}$$

where $Q(\omega^{i})$ is the matrix representation of the quaternion:

(9) $$Q\left(\omega^{i}\right)=\frac{1}{2}\left[\begin{array}{cc} 0 & -\omega_{j}^{iT}\\ \omega_{j}^{i} & -S(\omega_{j}^{i}) \end{array}\right]$$

The authors also assume that human limb acceleration is bounded and averages to zero over a certain amount of time, hence they propose to model the angular velocity as exponentially decaying over time:

(10) $${\dot{\omega }}_{k}^{i}=-\frac{1}{t_{k}}\omega_{k}^{i}\;k\in \left\{x,y,z\right\}$$

where $t_{k}$ is a parameter of the algorithm that determines the time horizon within which $\omega_{k}$ averages to zero. These two methods for estimating $q^{i}$ are fused by means of an EKF in which the state vector is

(11) $$X^{i}=\left[\begin{array}{c} \omega^{i}\\ q^{i} \end{array}\right].$$

The process model between steps *j* and j+1 is derived from Equations (8) and (10):

(12) $$X_{j+1}=\left(\Phi_{j}T+I_{7}\right)X_{j}+w_{j}$$

where $w_{j}$ is white noise in a 7-dimensional space and

(13) $$\Phi_{j}=\left[\begin{array}{cccc} -1/t_{1} & 0 & 0 & \\ 0 & -1/t_{2} & 0 & 0_{3,4}\\ 0 & 0 & -1/t_{3} & \\ & 0_{4,3} & & Q(\omega_{j}^{i}) \end{array}\right].$$

The measurement model is the identity as ${\tilde{\omega }}^{i}\approx \omega^{i}$ and $q^{i}$ measurement is $q_{m}^{i}$. The final estimation of $q^{i}$ is then used to reconstruct the pose of the links composing the human arm.

### 3.3. Method 3

**Figure 3:**
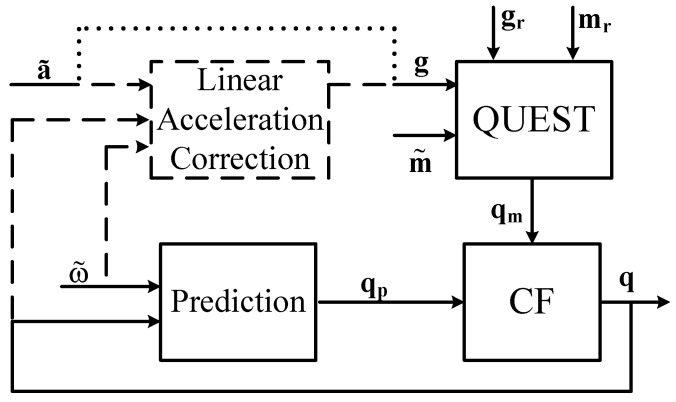
Diagram of method 3 for the *i*-th limb, the diagram represents one temporal slice of the motion reconstruction. Dashed lines apply to the *perfect* version only whereas dotted lines to the *pure* version only. Vectors $g_{r}$ and $m_{r}$ are the gravity and the magnetic field vectors represented in the *i*-th limb frame in a reference configuration, e.g., N pose.

The third method is presented in [58]. The attitude of the *i*-th limb with respect to the root is represented by the quaternion $q^{i}$. They assume to attach a mIMU sensor to each moving limb. The authors propose two versions of their method. In the first version that is called *pure* the linear acceleration is neglected. In the second version that is called *perfect* the authors model the human body as a kinematic chain that allows them to calculate the linear acceleration ${}^{l}a^{i}$ of each frame. Figure 3 summarizes the two versions of the method. The authors assume that the mIMU axes are aligned with the limb frames, thus having ${}^{i}m\approx {\tilde{m}}^{i}$, and they suppose to know all the parameters that are required to define the kinematic chain.

In both versions the authors propose a complementary filter in which the “high frequency” estimation of $q^{i}$, namely $q_{p}^{i}$ is obtained from the limb angular velocity $\omega^{i}$ as in Equation (8). The “low frequency" estimation of $q^{i}$, namely $q_{m}^{i}$ is obtained from ${}^{i}g^{i}$ and ${}^{i}m^{i}$ by means of the QUEST algorithm. Given $q^{i}$ estimation at time step *j*, the proposed CF computes

(14) $$q_{j+1}^{i}=\frac{1}{k}\left(q_{m}^{i}-q_{p}^{i}\right)+q_{j}^{i}$$

where *k* is a parameter that allows us to tune the filter.

The *pure* and the *perfect* filters differ in the $g^{i}$ that is provided to the algorithm:

(15) $$g^{i}=\left\{\begin{array}{cc} {\tilde{a}}^{i} & pure\;\mathrm{version},\\ {\tilde{a}}^{i}-{}^{l}a^{i} & perfect\;\mathrm{version}. \end{array}\right.$$

The authors associate a hierarchical model tree with the human kinematic chain so that one limb is the root and every other limb *i* has its parent *p*. Given the angular velocity of the *i*-th limb, ${}^{l}a^{i}$ is

(16) $${}^{l}a^{i}={}^{l}a^{p}+\left[S\left(\dot{\omega_{i}}\right)+S^{2}\left(\omega_{i}\right)\right]d_{i}^{p}$$

where ${}^{l}a^{p}$ is the linear acceleration of the *i*-th limb’s parent, S(ω) is defined in Equation (2), and $d_{i}^{p}$ is the position of *i*-th frame origin in the parent frame.

Finally $q^{i}$ is used to reconstruct each limbs pose according to the defined kinematic chain.

### 3.4. Method 4

The fourth selected method [45] has two main innovations with respect to the previous ones. First it embeds the kinematic constraint equations in the sensor fusion filter. Second it proposes a visual reference to aid magnetometers under severe magnetic disturbances. This method aims at tracking the upper body (but can be extended to the full body) by means of five mIMUs attached on the chest, the upper arms and the forearms. The mIMU on the chest is also provided with a camera (CmIMU) that tracks the markers placed on the user’s wrists. The authors consider a five degree of freedom (DoF) model for each arm and also model the shoulder motion accounting for the scapulohumeral rhythm [115]. The resulting kinematic chain is rooted in the chest and organized as a hierarchical tree.

**Figure 4:**
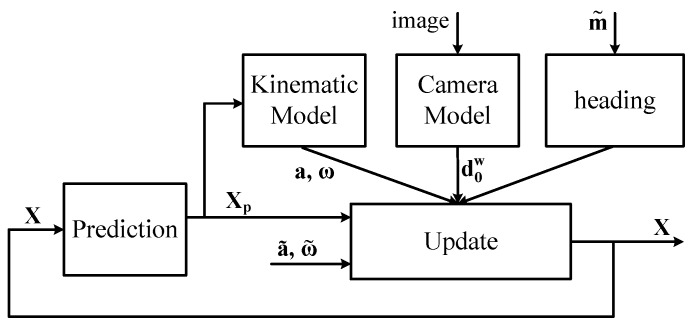
Diagram of the method 4, the diagram represents one temporal slice of the motion reconstruction.

The method is based on three loosely coupled EKFs, the first returns the trunk orientation given the mIMU signals, the latter two estimate the shoulder (three DoFs) and elbow (two DoFs) joint angles $q_{1},\ldots ,q_{5}$ of each arm based on the mIMU signals, the trunk orientation and the wrist position are obtained from the camera image. Figure 4 summarizes the components of the EKF that estimates the arm motion given the trunk orientation.

The state of these EKFs is $X={\left[q_{1}\;{\dot{q}}_{1}\;{\ddot{q}}_{1}\;\ldots \;{\ddot{q}}_{5}\right]}^{T}$, the process model is linear and it assumes constant angular acceleration between two time steps *j* and j+1, thus having for the *i*-th angle

(17) $${\left[\begin{array}{c} q_{i}\\ {\dot{q}}_{i}\\ {\ddot{q}}_{i} \end{array}\right]}_{j+1}=\left[\begin{array}{ccc} 1 & T & T^{2}/2\\ 0 & 1 & T\\ 0 & 0 & 1 \end{array}\right]{\left[\begin{array}{c} q_{i}\\ {\dot{q}}_{i}\\ {\ddot{q}}_{i} \end{array}\right]}_{j}+w^{i}\;i=1,\ldots ,5$$

The authors then propose a calibration procedure to relate the state X to the available measurements. Assumed that the mIMUs sit on the frames of the limbs, the orientation of each mIMU frame with respect to the related link frame is represented by the rotation matrix $R_{i}^{ui}$ that is obtained through this procedure as well as the position of the CmIMU with respect to the shoulder joint center. The other link lengths are gathered from anthropometric tables. Given $R_{i}^{ui}$, the orientation of each mIMU with respect to the root is $R_{0}^{ui}$ and the mIMU measurements are

(18) $$\begin{array}{cc} {\tilde{a}}^{i} & =R_{i}^{ui}\left({}^{i}a^{p}+{}^{i}g\right)+w_{a}\\ {\tilde{\omega }}^{i} & =\mathrm{vex}\left(R_{0}^{ui}{\dot{R}}_{0}^{ui}\right)+w_{o}\\ {\tilde{m}}^{i} & =R_{0}^{ui}m^{0}+w_{h} \end{array}$$

where vex(S(ω))=ω being S(ω) defined in Equation (2), $a^{p}$ is the acceleration of the link hosting the *i*-th mIMU, and $w_{j}$ are white noises. The latter measurement equation is only partially used and reduced to the heading direction. A further measurement equation relates the position of the wrist to the wrist position estimated from the camera:

(19) $${}^{c}{\tilde{d}}_{0}^{w}=\frac{1}{d_{0_{z}}^{w}}\left[\begin{array}{c} \begin{array}{c} d_{0_{x}}^{w}\\ d_{0_{y}}^{w} \end{array} \end{array}\right]+w_{c}$$

where $d_{0}^{w}$ is the wrist position and $w_{c}$ is white noise. The measurement equations are then grouped as

(20) Z=h(X)

that is linearized to obtain the observation matrix

(21) $$H=\frac{\partial h}{\partial X}{|}_{X_{j+1,j}}$$

This method directly provides the poses of all limbs.

### 3.5. Method 5

The last selected method [47] has two main differences with respect to the previous ones. First, it does not rely on the linearization of nonlinear equations, but it exploits the unscented transformation to cope with non-linearities. Second, it proposes refinements in the kinematics of the upper body and exploits a nonlinear sensor fusion algorithm to cope with nonlinear models. The method aims at upper limb motion tracking. Each of the clavicles, upper arm and forearm is provided with a mIMU. Taken the chest as root, a seven DoFs hierarchical kinematic model of each arm was developed according to the Denavit Hartenberg convention.

The sensor fusion technique of this method is an Unscented Kalman Filter in which the state vector is $X={\left[q_{1}\;{\dot{q}}_{1}\;{\ddot{q}}_{1}\;\ldots \;{\ddot{q}}_{5}\right]}^{T}$ and the process model is the same as Equation (17).

The authors propose a calibration procedure to gather the parameters needed to relate the state *X* to the measurements. The orientation of each mIMU frame with respect to the related link frame is represented by the rotation matrix $R_{i}^{ui}$, whereas its translation with respect to the parent frame is measured to obtain the homogeneous matrix $A_{i}^{ui}$ that fully refer the mIMU frame to its parent’s one. Given that the *s*th mIMU is attached to the *i*-th frame whose parent frame is *p*, the measurements model is:

(22) $$\begin{array}{cc} {\tilde{a}}^{s} & =R_{p}^{s}\;a^{p}+\left[S\left({\dot{\omega }}^{p}\right)\;+S{\left(\omega^{p}\right)}^{2}\right]r_{p}^{s}+R_{0}^{s}\;{}^{0}g+w_{a}\\ {\tilde{\omega }}^{s} & =R_{p}^{s}\left(\omega^{p}+{\dot{\vartheta }}_{p+1}z_{0}\right)+w_{o}\\ {\tilde{m}}^{s} & =R_{0}^{s}m^{0}+w_{h} \end{array}$$

where $R_{p}^{s}$ is the rotation matrix from the parent frame to the sensor frame, $z_{0}={\left[0,0,1\right]}^{T}$ vector, and $r_{p}^{s}$ is the position of sensor frame relative to parent in sensor frame. The measurement equations are then grouped as Equation (20). In this case the function h is not linearized, but it is used for the unscented transformation that provides the measurement estimation based on the state prediction. As for method 4, the state already provides he pose of each limb.

## 4. Comparison

### 4.1. Experimental Setup

**Figure 5:**
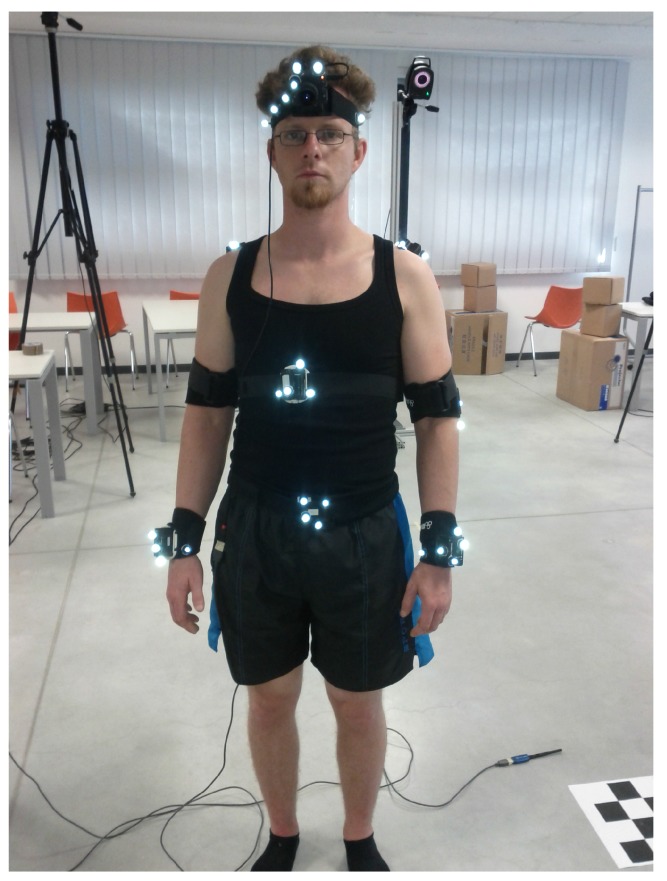
Experimental setup showing markers that were placed on the anatomical landmarks and on the 9 mIMUs.

Selected methods were compared to each other using OMC. Ground truth data were obtained from the Vicon (OMG plc, Oxford, UK) OMC system while tracking a healthy 28 years old male that was equipped with the mIMUs Colibri mIMUs from Trivisio Prototyping GmbH, sampled at 100 Hz), the CmIMU (Firefly MV color camera from PointGrey with diagonal field of view of 140 deg, sampled at 12.5 Hz, hardware synchronized with the mIMUs), and markers on the anatomical landmarks. After holding N-pose and T-pose as calibration procedure, he was asked to perform several movements that involved one functional degree of freedom at a time, namely elbow flexion/extension ($E_{\mathrm{FE}}$), forearm pronation/supination ($E_{\mathrm{PS}}$), shoulder flexion/extension ($S_{\mathrm{FE}}$), shoulder abduction/adduction ($S_{\mathrm{AA}}$), and shoulder internal rotation ($S_{\mathrm{IR}}$). The participant gave his informed consent for inclusion before he participated in the study. The study was conducted in accordance with the Declaration of Helsinki, and the protocol was approved by the Ethics Committee of Scuola Superiore Sant’Anna (Delibera n. 1/2017). The setup of the experiment is shown in Figure 5.

The joint motion reconstruction from optical data is based on the following kinematic model: the chest was considered as a steady rigid body. Shoulder was modeled as a spherical joint, so that the humerus has three rotational DoFs with respect to the chest. The forearm was considered to have two DoFs with respect to the humerus, i.e., the flexion-extension and pronation-supination functional DoFs. The Vicon Nexus^®^ software (Oxford Metrics, Oxford, UK) allowed us to use this kinematic model for offline adjustment of the marker positions. The marker on the acromions served to capture the shoulder joint center, that is assumed to be 4.5 cm under the acromion in the $Z_{v}$ direction.

Each sequence lasted at least 10 s. Wrist position was used to assess the methods. Moreover, each sequence of movements includes a pair of repetitions performed at higher speed to test how methods’ performance varies as the linear acceleration increases.

Captured data include the markers positions in the Vicon reference system $\tau_{v}$, the mIMU signals in the respective sensor reference systems, and the images gathered by the CmIMU in the camera reference system. All the data are synchronized and gathered at 100Hz. IMU and OMC data were manually synchronized by exploiting the transitions from static postures to motion. This synchronization method may introduce a time misalignment in the data which accounts for up to 3 samples (variations in the data are sufficiently clear to identify onset of motion). This means that the maximum misalignment cannot exceed 30 ms. The dataset used for the comparison is available in Zenodo (https://zenodo.org/) and it can be found through the digital object identifier (DOI) of this paper.

#### 4.1.1. Data Alignment for the Comparison

The comparison of the estimated positions (joints and end effectors) against the OMC based ones requires to represent both in a common reference system. OMC data is available in a frame $\tau_{v}\equiv \left(O_{v},x_{v},y_{v},z_{v}\right)$ that is defined during the OMC system calibration. Since the chest frame $\tau_{0}$ was the root for the IMU based estimation, all these data were available in $\tau_{0}$. Once decided to represent all the body frames in $\tau_{v}$, the rigid transformation between the global frame $\tau_{v}$ and the root IMU frame $\tau_{0}$ is sufficient for the comparison. The homogeneous transformation matrix

(23) $$A_{0}^{v}=\left[\begin{array}{cc} R_{0}^{v} & {}^{v}d_{0}^{v}\\ 0 & 1 \end{array}\right]$$

represents such a transformation, where $R_{0}^{v}$ is the rotation matrix that aligns $\tau_{0}$ axes with $\tau_{v}$ ones, and ${}^{v}d_{0}^{v}$ is the position vector of the $\tau_{0}$ origin. Since in data capturing there was not enough information to calculate $A_{0}^{v}$, it was decided to estimate it for each method. Let $\tilde{X}=\left[{}^{v}{\tilde{x}}_{1},\cdots ,{}^{v}{\tilde{x}}_{n}\right]$ be a *n* samples set of optical captured positions and $\tilde{Y}=\left[{}^{0}{\tilde{y}}_{1},\cdots ,{}^{0}{\tilde{y}}_{n}\right]$ the corresponding estimated positions. Let then ${}^{v}y$ and ${}^{0}x$ be the captured and reconstructed positions in the reference configuration, i.e., the N-pose for the present evaluation. If we consider two new sets of samples, namely *X* and *Y*, obtained from $\tilde{X}$ and $\tilde{Y}$ as

(24) $$\begin{array}{cc} x_{j} & ={\tilde{{}^{v}x}}_{j}-{}^{v}x\\ y_{j} & ={\tilde{{}^{0}y}}_{j}-{}^{0}y. \end{array}$$

then

(25) $${}^{v}d_{0}^{v}=0.$$

The rotation matrix $R_{0}^{v}$ is calculated to minimize the reconstruction error, for any method *m* and a reference *r* the quality *Q* can be computed as

(26) $$R_{0}^{v}=\underset{R_{0}^{v}}{\mathrm{argmin}}\sum_{j=1}^{n}{∥\left(y_{j}-R_{0}^{v}x_{j}\right)∥}^{2}.$$

Finally, the samples set $Z=[{}^{v}x_{1},\cdots ,{}^{v}x_{n}]$ obtained as

(27) $$Z=R_{0}^{v}Y$$

can be compared against X.

This method underestimates the absolute error of each method, but it provides a fair comparison between the methods. The quality of a set of body tracking techniques can then be evaluated against the OMC dataset by comparing the joint and end-effector points with the reference method.

#### 4.1.2. Performance Indices

Aligned mIMU and OMC data are then used to calculate the performance measures that we introduced in Section 2.2.4 and hence compare the algorithms. Given two random variables *X* and *Z* each sampled with *N* samples, the following indices will be used for this purpose:

1. Accuracy:
  (28) $$E=\frac{1}{N}\sum_{i=1}^{N}∥Z_{i}-X_{i}∥$$
2. Correlation:
  (29) $$C=\mathrm{cor}\left(X,Z\right)=\frac{\mathrm{cov}(X,Z)}{\sqrt{\mathrm{var}(X)\mathrm{var}(Z)}}$$

### 4.2. Experimental Results

**Figure 6:**
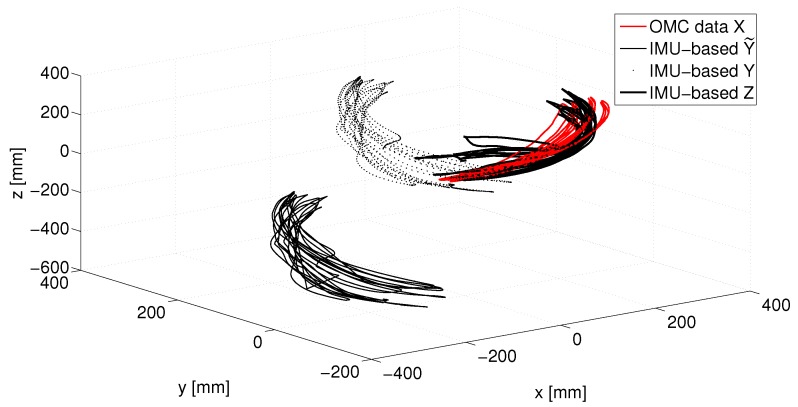
Alignment procedure for the mIMU-based data. mIMU-based estimated (*Y*), translated ($\tilde{Y}$) and aligned (*Z*) are reported along with OMC data (*Z*).

Data gathered from the mIMUs provided the input for the methods reported in Section 3 to reconstruct the arm kinematics. The parameters of each method’s filter were selected to optimize the method performance in terms of stability and accuracy. To enable the comparison of the methods, OMC and mIMU-based position estimation were aligned according to the method reported in Section 4.1.1. Figure 6 shows how the mIMU-based data are first translated to match OMC data in N-pose and then rotated to obtain the best alignment with OMC data.

**Figure 7:**
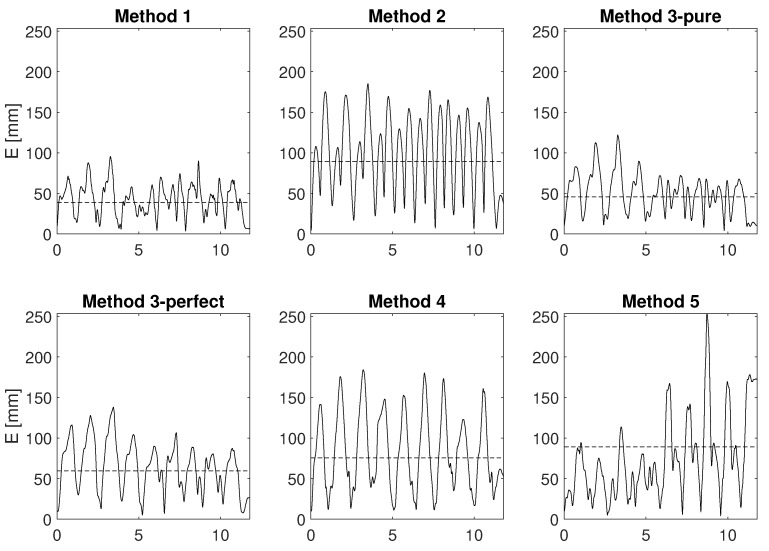
Error on the wrist motion reconstruction for the E${}_{\mathrm{FE}}$ functional motion trial. On the *x*-axis the seconds elapsed since the trial beginning are shown. Dotted lines represent average error in the trial.

Figure 7 refers to the $E_{\mathrm{FE}}$ functional motion and shows how the error E (see Equation (28)) evolves over time.

After these examples we report the values of the error and of the correlation that the different methods scored. For the comparison of the methods three functional movements were selected, namely $E_{\mathrm{FE}}$, $S_{\mathrm{FE}}$, $S_{\mathrm{AA}}$. The first movement allows us to assess how the methods behave when only one joint of the kinematic chain as well as only one mIMU moves. The latter two involve the motion of two mIMUs. With respect to the $E_{\mathrm{FE}}$ motion, in the $S_{\mathrm{FE}}$ and $S_{\mathrm{AA}}$ motions the estimation provided by methods that use the kinematic chain is likely to differ more from the other methods’ estimation. The average of *E* and *C* in the three trials are reported in Table 1.

**Figure 8:**
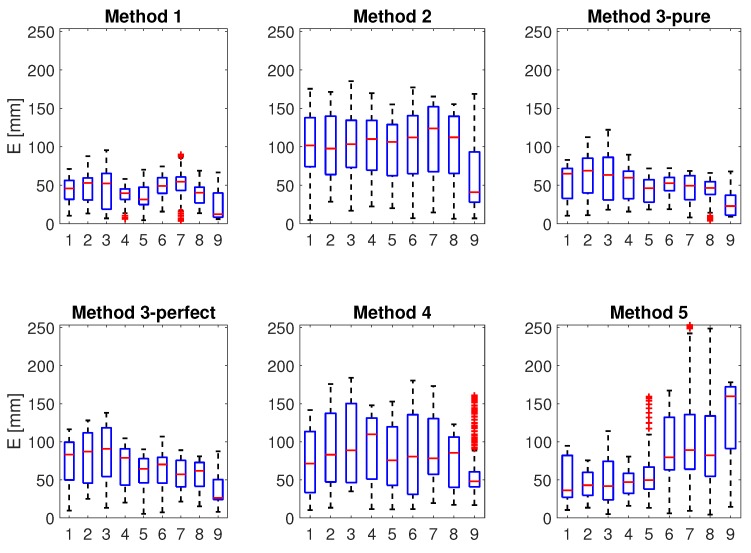
Error on the wrist motion reconstruction for each period of the E${}_{\mathrm{FE}}$ functional motion trial. The boxplots show the median of *E* along with the 25th and 75th percentiles. Whiskers extend to 1.5 times over the interquartile range.

To obtain a more detailed insight of the methods’ performance, the $E_{\mathrm{FE}}$ functional motion was further studied. It was divided into cycles: cycles 1–7 were carried out at a slower speed, whereas the latter two were carried out at a higher speed. Figure 8 shows how the error E is distributed within the cycles of the $E_{\mathrm{FE}}$ trial, whereas Table 2 reports the average of E and C (see Equation (28)) for the same cycles.

### 4.3. Discussion

The methods can now be compared according to the indices that were proposed in Section 4.1.2. Before comparing the methods, we see from Figure 7 that for all the methods the error varies periodically with time. This time error may be due to residual error of mIMU-OMC data alignment. However, each of the methods may have other source of error related to biomechanical constraints: lack of kinematic constraints (methods 1, 2 and 3 pure) and too rigid constraints (method 3 perfect, 4 and 5) are all suitable candidates for periodic errors in the estimation of a periodic motion.

#### 4.3.1. Accuracy

The first measure that we proposed is *accuracy*, as obtained via *E*. Clearly, the lower *E* the more accurate the method is. Accuracy is a measure of reliability in accurate position estimation, accuracy is needed when an absolute measure of the position is required, for example in the analysis of motion or to provide force rendering when interacting with a Virtual Environment. From Table 1 we see that method 1 is generally the most accurate and method 4 is also comparable. We note that the accuracy gap between methods 1 and 4 is smaller for $S_{\mathrm{AA}}$ and $S_{\mathrm{FE}}$ movements, being method 4 more accurate for the latter movement. This partially supports the finding that imposing a kinematic chain in the motion estimation improves the estimation when the measurements of mIMUs on different links are affected by the same joint variable. This hypothesis is further supported when methods 1, 2 and 3-pure are compared. Differently from methods 3-perfect, 4 and 5, the first two methods do not take into account the kinematics of the arm. The latter has a lower accuracy in the $E_{\mathrm{FE}}$ motion, but has a better accuracy for the motions $S_{\mathrm{AA}}$ and $S_{\mathrm{FE}}$ (except for method 3-perfect in the $S_{\mathrm{AA}}$ motion).

#### 4.3.2. Correlation

*Correlation* *C* is the second measure that is considered. Correlation indicates whether the estimated position follows the real pattern of the performed movement. A good correlation of the estimated human motion with the real movement suffices to teleoperate a remote robot. In facts, in this case it is anyhow needed to map the operator motion to the robot kinematics, and what matters is that this map does not vary along time. In other words, even if accuracy is poor, the human who teleoperates the robot can easily adapt motion of his arm to be able to control the robot unless there is a good correlation between the performed motion and the method’s estimate. When correlation is poor, human has to adapt the motion of the arm and eventually perform unnatural motions to control the robot (e.g., activate more DoFs to obtain a simple elbow motion). From the point of view of the correlation, Table 1 shows that the best performing methods are 4 and 5.

#### 4.3.3. Fast Motion

One of the differences between the methods is the use of the kinematics of the human arm, in particular of the linear acceleration of the limbs. A reasonable hypothesis is that methods 3-perfect, 4 and 5 perform better with respect to the others when the motion of the limbs is fast and the speed changes quickly. This condition should make linear acceleration of the limbs play a bigger role. In our case, this role is enhanced by mounting the mIMUs far from the parent joint. However, looking at Figure 8 and at Table 2, there is no significant difference in the variation of the accuracy between slow cycles (1 to 7) and fast cycles (8 and 9). Similarly, Table 2 shows that correlation does not improve between cycles 1–7 and 8–9 for methods 1,2,3-perfect and 5, whereas there is a small improvement for method 4. These results suggest that the linear acceleration of the limbs plays a minor role with respect to gravity, as assumed by several models. However, this aspect should be further investigated with specific motions in which gravity plays a minor role to identify limb’s orientation.

#### 4.3.4. Sources of Error

As a final remark of the discussion, the accuracy and the correlation that were obtained are generally comparable or worse with respect to the literature. However, apart from possible suboptimal tuning of the methods’ parameters, possible sources of error that can explain our results

- Knowlegde of human parameters (i.e., arm length). This source of error can be minimized by including human parameters in the estimation e.g., [51]
- Body to mIMUs calibration. Although the calibration procedure that we carried out suffices to determine the orientation of the mIMUs, uncertainties in the position of mIMUs with respect to their parent is still subject to assumptions. Also the effects of this source of error can be reduced by a proper calibration and by taking into account the sensor poses in the sensor fusion technique.
- Time alignment of OMC data with mIMU data. OMC and mIMU-based data are manually done based on a known motion from a steady condition. However, the effects of misalignment are much smaller than the error we have reported.
- Preprocessing of data. Here we tested only the reported algorithm, not considering possible filtering on mIMU data. For example, having a hard magnetic calibration, it would be possible to handle bad data with distorted magnetic field.

## 5. Conclusions

After introducing the reader to the main issues of IHMT, relevant methods from the literature were reported. Analysis of the literature revealed that several approaches perform similarly for IHMT. However, optimization-based methods seem to have the potential to bring substantial improvements to IHMT. Currently methods and solutions for lower limbs tracking such as ZUPT have not been widely applied to full body tracking yet. These methods are often capable of accurate estimation even during long walk trials. Therefore their combination with their upper limbs counterparts may improve accuracy and reduce drift of full body IHMT also when walking.

Five methods that span the different techniques used for IMU data sensor fusion were presented and analysed in depth, and an evaluation of these methods was proposed based on accuracy and correlation with OMC data. Results showed that method 1 is the best performing for accuracy followed by method 4, which is the best in terms of correlation. We hence advise to use method 1 for attitude estimation and for navigation purposes. Instead we consider 4 the best method for robot teleoperation. Motion speed analysis provided minor results, possibly due to the choice of movements that make gravity play a dominant role in limb attitude estimation.

## Figures and Tables

**Table 1:** Accuracy and correlation of the methods’ estimation. Error *E* and correlation *C* are calculated according to Equation (28) for elbow flexion-extension $E_{FE}$, shoulder flexion-extension $S_{FE}$, and shoulder abduction-adduction $S_{AA}$. For each performance index (i.e. each column), bold values highlight methods that performed better.

	E${}_{\mathrm{FE}}$		S${}_{\mathrm{FE}}$		S${}_{\mathrm{AA}}$	
Method	E (mm)	C	E (mm)	C	E (mm)	C
1	**38.8**	0.86	108.9	0.46	**66.0**	0.66
2	89.2	0.77	121.4	0.86	243.8	0.36
3-pu.	45.7	0.84	122.86	0.46	156.0	0.59
3-pe.	59.7	0.84	100.4	0.50	272.2	0.60
4	75.7	**0.91**	**82.7**	0.86	86.0	**0.73**
5	89.2	0.77	214.4	**0.89**	125.4	0.66

**Table 2:** Accuracy and correlation of the methods’ estimation. Error *E* and correlation *C* are calculated according to Equation (28) for each period of the E${}_{\mathrm{FE}}$ motion. For each cycle, bold values highlight methods that performed better. The amount of bold values per column allows the reader to have an impression of what methods performed better.

		Method					
**Cycle**	**Index**	**1**	**2**	**3-Pure**	**3-Perfect**	**4**	**5**
E (mm)	1	**43.7**	102.8	53.8	73.4	72.3	51.3
	2	49.9	100.3	64.2	79.9	90.7	**44.8**
	3	**46.6**	103.4	63.1	84.0	98.3	49.4
	4	**37.7**	102.8	53.3	68.1	92.1	47.7
	5	**35.3**	97.1	44.5	57.6	79.0	59.6
	6	**49.4**	102.8	51.3	64.1	85.7	89.6
	7	50.4	108.4	**45.9**	57.5	90.5	101.3
	8	**38.9**	100.2	44.7	56.3	76.0	97.8
	9	**23.6**	60.9	27.8	36.5	59.0	136.9
C	1	0.94	0.73	**0.96**	0.92	0.94	0.93
	2	**0.94**	0.77	0.91	0.87	0.92	0.85
	3	**0.96**	0.75	0.91	0.82	0.95	0.81
	4	0.95	0.72	0.96	0.92	**0.96**	0.76
	5	**0.95**	0.78	0.93	0.89	0.94	0.87
	6	0.93	0.81	**0.95**	0.92	0.92	0.82
	7	0.83	0.77	0.84	0.85	**0.91**	0.76
	8	0.88	0.74	0.86	0.89	**0.95**	0.71
	9	0.87	0.86	0.92	0.93	**0.95**	0.94

## Appendix A. Summary of Presented Methods

A summary of the methods presented in Section 2 is reported in Appendix A in Table A1. Methods are sorted in chronological order and described according to the following categories:

**Ref**: reference to the article where the methods described.

**Year**: publication year.

**Body**: this category describes which part(s) of the body are targeted by the method Application: some methods are connected by the authors to one ore more applications. Most of the times this link is made explicit in the introduction. This category reports the application(s) extracted from the article. They span from computer graphics to robotics and medical applications.

**Target**: this category describes the physical quantities that are targeted by the method in relation to the body parts to be tracked. For example, when the body parts to be tracked are upper limbs, arm’s orientation and position are possible targets. The word “attitude” is referred to methods that are not referred to a specific part of the human body but to a generic rigid body.

**Focus**: Most of the cited articles focuses on the description of the IHMT method. However, some articles focus on the assessment of some methods, or on their application. In these cases fewer or no details about the IHMT method are reported. Calibration and handling of magnetic disturbances are also focuses of some of the articles reported in Section 2.

**Sensors**: List of the sensors required to apply the method described in the article. Each sensor is preceded by the numbers of units that are used.

**Kinematics and Constraints**: This category lists the kinematic representation(s) that was (were) chosen in the method and the biomechanical constraints that were used. Orientation is often represented as a quaternion or a rotation matrix which is a function of three independent variables. Exponential maps have also been used to represent orientation. When a kinematic chain is used, position and orientation may be obtained through the joint variables. This is the case when “kinematic chain” is reported without other kinematic representation specifications (e.g., “quaternion orientation”). “Kinematic chain” also indicates that joints are used either to impose constraints to motion or to reconstruct limbs’ pose. This category reports also the use of free segments modelling. Other constraints specifications (e.g., hinge, soft or hard constraints) are specified for some methods. This category is linked to Section 2.2.1.

**Parameters**: Kinematic chain, constraints and sensor fusion technique require to set and tune some parameters. These parameters are grouped and reported in this category. Lengths (e.g., arm length), orientations (e.g., mIMU’s orientation with respect to the link it is attached to), Kalman Filter parameters (e.g., covariance matrices) are parameters that many models require.

**Sensor Fusion Technique**: The mathematical method(s) that was (were) used is reported in this category. Kalman Filters, Complementary Filters and Optimization are the most popular possibilities that are reported. However, some methods use other tools such as inertial navigation systems and probabilistic graphical models. This category is linked to Section 2.2.2.

**Calibration**: When one or more calibration procedures are required to obtain the parameters needed for the method, these are here summarized. Most of the methods include static calibration, whereas other requires more complex procedures which include motion of specific articulations. This category is linked to Section 2.2.3.

**Validation**: Validation methods are reported in this category, the number of participants and the source of ground truth data is reported.

**Measure**: Quantitative validation of the methods requires the definition of variables to be assessed. In many cases these variables replicate the target of the method, in other cases they are a subset of the variables need for tracking of the declared targets.

**RMSE**: Root mean square error of the variables reported as measures for the validation when compared to ground truth data. Angular variables are reported in degrees whereas position are reported in millimetres. Drift are reported in meters per seconds.

**Correlation**: Correlation of estimated variables with ground truth data as reported in the referenced article.

The **Notes** column completes the description of each method with a few details specific of the method. Some formulas are worth to be mentioned:

**Sens align link** refers to the assumption that a sensor’s frame is supposed to be aligned to the frame of the body it is attached to; **Sens sitting link** is used referring to the situation of a (m)IMU attached to a human limb. In this case the the limb is supposed to be a beam and the formula refers to the assumption that the sensor’s frame origin is a point of such a beam; **Bias est** refers to models in which sensors’ biases are among the variables that are estimated;

**Lin acc noise** is referred to methods in which linear acceleration of the sensor is considered to be noise;

**Show plot** refers to method for which RMSE and/or correlation are not reported as figures in the text on in tables but a plot of such quantities is available.

### Appendix A.1. Description of Abbreviations

In Table A1 some abbreviations are used to make the table more compact. These abbreviations are listed according to the previously defined categories and described in Table A2:

**Table A1:** Summary of IHMT methods.

Ref.	Year	Body	Application	Target	Focus	Sensors	Kinematics & Constraints	Parameters	Sensor Fusion Technique	Calibration	Validation	Measure	RMSE	Correlation	Notes
[30]	1999	attitude	computer graphics	link ori	method	1 acc, 1 gyro, 1 mag	quat ori	CF gain	CF, GN opt	no	1, tilt table	roll	1.0	-	initial condition study, 120 s valid trial, lin acc negl
[31]	2001	attitude	computer graphics	link ori	method	1 acc, 1 gyro, 1 mag	quat ori	CF gain	CF, GN opt	-	1, tilt table	quat compar	-	-	initial condition study, 25 s valid trial
[10]	2004	attitude	computer graphics	link ori	method	1 acc, 1 gyro, 1 mag	rot mat	CF gain	CF	-	1, robot EE	roll, pitch, yaw	-	0.7–0.87	drift, 12s valid trial, omni phantom
[32]	2004	upper limbs	-	arm ori forearm ori wrist pos	method	2 acc, 2 gyro, 2 mag	rot mat	link len, KF params	KF, QUEST	-	1, hor line vert line	wrist pos, shoulder abd, elbow fle	-	-	lin acc negl, sens align link, sens sitting link, 25s valid trial, show plot
[57]	2004	full body	teleoperation	link ori	application	14 acc, 14 gyro, 14 mag	kinematic chain, quat ori	CF gain	CF like	-	robot EE	-	-	-	sens sitting link, plot traj, valid teleop robot
[95]	2005	upper limbs, lower limbs	medical	link ori	method	1 acc, 1 gyro	rot mat	KF params	KF	static	2, OMC	pelvis incli trunk incli forearm incli pelvis drift trunk drift forearm drift	pelvis incli 1 trunk incli 2 forearm incli 3 pelvis drift 0.6 trunk drift 0.4 forearm drift 0.4	-	drift modeled, sens align link
[84]	2005	attitude	-	link ori	mag comp	1 acc, 1 gyro, 1 mag	rot mat	KF params	KF, lin acc err, mag err	static	1, box, 1, OMC	ori err sta ori err dyn	ori err sta 0.6 ori err dyn 2.7	-	linear acc noise,
[75]	2005	attitude	-	link ori	method	1 acc, 1 gyro, 1 mag	quat ori	CF gain	CF	-	mech platform	link ori	-	-	bias est, 60s trial, show plot
[36]	2005	lower limbs	medical: locomotion	link ori link pos	method	1 acc, 1 gyro	rot mat	-	acc double int	static dynamic 6 static poses 6 known traj speeds	1, OMC	link ori link pos	link ori 0.8–1.3 link pos 22–26	-	sens sitting link, sens align link, 4 s localization valid tasks
[88]	2006	lower limbs	-	shank ori	method	2 2d acc, 1 gyro	knee hinge	CF gain	CF like	static, n pose like	8, OMC	shank thigh	shank 0.74–0.78 thigh 1.42–1.69	0.999	motion limited sagittal
[96]	2006	lower limbs	medical: knee function analysis post cruciate ligament lesion	knee fle knee abd knee rot	application	2 gyro	-	-	gyro int	-	5, US	knee fle knee abd knee rot	knee fle 4.4 knee rot 2.7 knee abd 4.2	-	target ROM, 30m walk valid trial
[97]	2006	attitude	computer graphics	link ori	method	1 acc, 1 gyro, 1 mag	quat ori	KF params	EKF, QUEST	-	1, tilt table	roll pitch yaw	2.0–9.0	-	initial condition study, 25 s valid trial
[50]	2006	upper limbs	computer graphics	shoulder ori shoulder pos arm ori arm pos forearm ori	method	1 2d acc, 1 1d gyro, mech track wrist pos	kinematic chain, quat ori	link len, KF params	KF, GN opt	-	1, OMC	shoulder ori elbow ori wrist ori	<OMC prec	-	sens align link, sens sitting link, bias est
[38]	2006	attitude	medical	link ori	method	1 acc, 1 gyro, 1 mag	quat ori	KF params	EKF	-	1, OMC	quat err roll pitch yaw	quat err 4.57 roll 1.31 pitch 1.40 yaw 4.13	-	adaptive covariance, bias est, ZUPT like sens inline calib, 120s free movements valid trial
[54]	2007	upper limbs	medical: monitoring, neuromuscular disorders	elbow angle	method	2 acc, 2 gyro	elbow hinge	KF params	KF	dynamic	1, OMC	elbow fle	elbow fle 8–25	-	static plus pro mov calib, variation R, 130s daily activities valid trial
[99]	2007	full body	-	arm ori forearm ori hand ori pelvis ori thigh ori shank ori	method	1 acc, 1 gyro, 1 mag magnetic coil	rot mat	link len, KF params	INS, EKF	-	6, OMC	trunk ori arm ori thigh ori trunk pos arm pos thigh pos	trunk pos 4.8–5.6 arm pos 5.0–7.9 thigh pos 8.6 trunk ori 2.4–3.0 arm ori 2.3–3.1 thigh ori 3.2	-	sens align link, sens sitting link, 30s walking valid trial
[14]	2007	full body	computer graphics, sport	arm ori forearm ori hand ori trunk ori pelvis ori thigh ori shank ori	method	18 acc, 18 gyro, 18 US	kinematic chain, quat ori	link len, KF params	EKF	static, rest pose	1, OMC	head ori arm ori thigh ori	head ori 5.7 arm ori 8.0 thigh ori 6.6	-	sens sitting link, 30s valid trials, drift observed
[92]	2008	lower limbs	medical	knee fle knee abd knee rot	calibration	2 gyro	-	-	gyro int	static, n pose, dynamic hip abd	10, MAG	knee fle knee abd knee rot	knee fle 1.5 knee rot 1.6 knee abd 1.7	knee fle 1.0 knee rot 0.95 knee abd 0.86	30m walk valid trial
[37]	2008	lower limbs	medical: gait	hip fle hip abd hip rot knee fle knee abd knee rot ankle fle ankle abd ankle rot	calibration	2 acc, 2 gyro, 2 mag	-	-	-	static	6, OMC	hip fle hip abd hip rot knee fle knee abd knee rot ankle fle ankle abd ankle rot	hip fle 3.0 hip abd 3.6 hip rot 4.5 knee fle 2.4 knee abd 4.8 knee rot 9.4 ankle fle 1.2 ankle abd 5.5 ankle rot 21.7	-	calibration from 6 participants
[48]	2008	upper limbs	medical	scapula pro scapula ret scapula ele shoulder fle shoulder abd shoulder rot elbow fle elbow pro	calibration	4 acc, 4 gyro, 4 mag	-	-	-	static	1, OMC	scapula pro scapula ret scapula ele shoulder fle shoulder abd shoulder rot elbow fle elbow pro	0.2—3.2	-	
[43]	2008	attitude	-	link ori	method	1 acc, 1 mag	quat ori		FQA	-	1, tilt table	roll, pitch, yaw	-	-	sens align to link
[76]	2008	attitude	-	link ori	method	1 acc, 1 gyro, 1 mag	quat ori	CF gain	CF	-	robot EE	link ori	-	-	bias est, 60 s valid trial, show plot
[63]	2009	lower limbs	-	shank ori thigh ori knee fle	method	2 acc, 2 gyro	knee hinge	KF params	KF	static, n pose like	7, OMC	shank ori thigh ori knee fle	shank ori 0.4–4.7 thigh ori 0.4–1.5 knee fle 0.7–3.4	-	
[98]	2009	lower limbs	-	hip ab hip fle knee fle	method	4 acc, 4 gyro	knee hinge		rot acc	-	8, OMC	hip abd hip fle knee fle	hip abd 3.96 hip fle 4.46 knee fle 3.73	0.91	limited motion to 80 deg, low speed valid trial
[103]	2009	lower limbs	medical	knee fle knee abd knee rot	calibration	2 gyro	-	-	gyro int	dynamic	8, MAG	knee fle knee abd knee rot	knee fle 8.1 knee rot 4.0 knee abd 6.2	knee fle 1.0 knee rot 0.85 knee abd 0.76	
[64]	2009	full body	computer graphics	head ori trunk ori pelvis ori arm ori forearm ori thigh ori shank ori	calibration, application	17 acc, 17 gyro, 17 mag	soft joints cardan euler helical joint coord sys	link len, KF params	KF	static, t pose, dynamic, axis rot, closed loop calib	-	-	-	-	three steps calib, closed loop calib
[94]	2010	lower limbs	medical: monitoring cerebral palsy	hip fle knee fle	calibration	8 acc, 8 gyro, 8 mag	-	-	-	static	9, OMC, 2, manual	ankle fle par knee fle par	1.4-1.8	-	manual measurement therapist
[58]	2010	full body	computer graphics	pelvis ori thigh ori shank ori foot ori	method	9 acc, 9 gyro, 9 mag	kinematic chain, quat ori	link len, sens pos, CF gain	CF, lin acc err	-	sim sens meas	pelvis ori thigh ori shank ori foot ori	pelvis ori 1.64 thigh ori 1.82–2.2 shank ori 1.81–2.02 foot ori 2.31–2.96	0.939 0.999	sens align link, sens sitting link, walking gait, running gait
[112]	2010	pose	-	link ori link pos	mag comp	1 acc, 1 gyro, 1 mag magnetic coil	rot mat	-	INS, EKF, mag sto model	-	static pos	-	-	-	
[114]	2010	full body	sport	link ori link pos	application	16 acc, 16 gyro, 16 mag	-	-	-	-	2, GNSS	link ori traj len	link ori 0.8–4.2 traj len 8	-	35 s pendulum valid trial, entire ski race
[67]	2010	upper limbs	-	pelvis pos pelvis ori trunk pos trunk ori arm pos arm ori forearm pos forearm ori	method	6 acc, 6 gyro, 6 mag	kinematic chain	link len sens pos	NR opt, inv kin	-	1, OMC	wrist pos	wrist pos 5	-	sens sitting link, sens align link, 180s valid trial, lin acc negl
[69]	2010	pose	localization	foot ori foot pos	method	1 acc, 1 gyro, 1 mag	rot mat	KF params	EKF, INS	-	1, known path	foot pos	foot pos 450–1350	-	bias est, lin acc est, ZUPT, ZARU, HDR, 100s valid trial 125m
[87]	2010	upper limbs	-	link ori link pos	method	2 acc, 2 gyro, 2 mag	kinematic chain, rot mat	KF params, link len	KF, CF like	-	8, OMC	wrist pos elbow pos wrist pos drift arm ori forearm ori	wrist pos 3–15 elbow pos 4–6 wrist pos drift 0.3 arm ori 2.04–2.06 forearm ori 2.16–2.41	wrist pos 0.95–0.97 elbow pos 0.95–0.98 arm ori 0.94–0.98 forearm ori 0.96–0.97	sens sitting link, sens align link, const lin acc, const ang vel, sens reloc, 30s square and circle valid trials, 100 s daily activities valid trials
[80]	2010	full body	-	link ori	method	-	-	KF params	KF	-	1, OMC	-	-	-	lowest point alg, const height ground, lin acc noise, show plot, drift visible, ZUPT
[18]	2010	full body	-	arm ori forearm ori hand ori pelvis ori thigh ori shank ori	method	1 acc, 1 gyro, 1 mag, mag coil	rot mat	link len, KF params	INS, EKF	-	6, OMC	trunk ori trunk pos thigh ori thigh pos arm ori arm pos	trunk ori 3.6–4.5 trunk pos 26–35 thigh ori 2.8–3.6 thigh pos 47–62 arm ori 2.8 arm pos 25	-	sens align link, sens sitting link
[45]	2011	upper limbs	industrial assembly	arm ori forearm ori wrist pos	method	5 acc, 5 gyro, 5 mag, Camera marker	kinematic chain, rot mat	KF params sens pos link len	EKF	static n pose, back bent	1, OMC	wrist pos	-	-	show plot, visual insp drift, 40 s valid trial
[41]	2011	full body	computer graphics, sport	arm ori forearm ori hand ori trunk ori pelvis ori thigh ori shank ori	method	10 acc, 10 gyro, 10 mag	kinematic chain exp map quat ori	link len, OPT params	OPT, VMF dist	static	1, MVN	5 links ori	7.3	-	sens sitting link, 20 s valid trials
[59]	2011	attitude	medical	link ori	method	1 acc, 1 gyro, 1 mag	quat ori	CF gain	CF like, OPT	-	1, OMC	roll pitch yaw	roll 0.581–0.625 pitch 0.497–0.688 yaw 1.073–1.110	-	sens sitting link, sens align link, average over 860 s valid trials
[40]	2011	attitude	medical	link ori	method	1 acc, 1 gyro, 1 mag	quat ori	KF params	EKF	-	1, OMC	roll pitch yaw	roll 1.75 pitch 1.96 yaw 5.46	-	sens sitting link, sens align link, adaptive covariance, bias est, 20 s valid trial, lin acc negl
[68]	2011	full body	medical	link ori link pos	application	1 acc, 1 gyro, 1 mag	quat ori	KF params, link len	EKF	static	1, MTX	roll pitch yaw	roll 1.62 pitch 0.8 yaw 6.06	-	sens sitting link, sens align link, adaptive covariance, bias est, 20 s valid trial, lin acc negl
[49]	2011	upper limbs	-	arm ori arm pos forearm ori forearm pos	method	2 acc, 2 gyro, 2 mag	kinematic chain, rot mat	KF params, link len	UKF	static, n pose	1, OMC	shoulder fle shoulder abd shoulder rot elbow fle elbow pro	shoulder fle 2.35 shoulder abd 0.877 shoulder rot 2.90 elbow fle 6.18 elbow pro 13.06	shoulder fle 0.999 shoulder abd 0.999 shoulder rot 0.997 elbow fle 0.887 elbow pro 0.964	DH, sens sitting link, sens fixed to limit soft tissue effect, lin acc negl
[39]	2011	upper limbs	computer graphics	arm ori arm pos forearm ori forearm pos	method	2 acc, 2 gyro, 2 mag	kinematic chain quat ori free seg	PF params, link len	PF	static, n pose	1, OMC	shoulder rot shoulder pitch shoulder fle elbow fle elbow pro	shoulder rot 3.23 shoulder pitch 1.32 shoulder fle 2.52 elbow fle 12.14 elbow pro 6.33	shoulder rot 0.999 shoulder pitch 0.999 shoulder fle 0.996 elbow fle 0.887 elbow pro 0.964	bias est, lin acc negl, sens sitting link, sens fixed to limit soft tissue effect
[53]	2011	upper limbs	-	arm ori forearam ori	method	2 acc, 2 gyro, 2 mag	rot mat	KF params link len sens pos sens ori	UKF	-	1, OMC	elbow fle elbow pro shoulder fle shoulder abd	-	elbow fle 0.89–0.92 elbow pro 0.93–0.96 shoulder fle 0.94–0.97 shoulder abd 0.91–0.94	DH, sens sitting link, sens align link, 5 s anat movements valid trials
[100]	2011	full body	-	link ori	assessment	9 acc, 9 gyro, 9 mag	quat ori	-	-	static, 12 poses	1, OMC	roll pitch yaw	rollIC 4.3–8 pitchIC 2.2–4.8 yawIC 2–11.4 rollSC 0.5–2.7 pitchSC 0.5–1.6 yaw 1.5–3.1	-	inter MIMU error, intra MIMU error, static valid trial, MTX proprietary KF
[77]	2012	upper limbs	-	arm ori forearm ori	method	2 acc, 2 gyro, 2 mag	kinematic chain, rot mat	link len, KF params	UKF	static, n pose	8, OMC	shoulder abd shoulder fle elbow fle elbow pro	shoulder abd 4.4 shoulder fle 5.5 elbow fle 6.5 elbow pro 5.5	shoulder abd 0.99 shoulder fle 0.98 elbow fle 0.98 elbow pro 0.95	DH, sens align link, sens sitting link, bias est, calib remove gyro bias, 12 s functional movements valid trials, 12 s daily actitvities trials
[81]	2012	full body	-	link pos link ori	mag comp	1 acc, 1 gyro, 1 mag	-	-	OPT	-	mech platform	mag magnitude mag center	mag magnitude 0.01 mag center 0.01	-	
[44]	2012	upper limbs	medical: rehabilitation	link ori link pos	method	2 acc	quat ori	opt params	NR opt	static	1, MTX	shoulder fle shoulder rot elbow fle elbow pro	shoulder fle 2.12 shoulder rot 4.78 elbow fle 3.7 elbow pro 3.16	-	motion limited sagittal, lin acc negl, sens sitting link, sens align link, static poses calib, 40 s trial sagittal plane
[66]	2013	upper limbs		link ori link pos	method	n acc, n gyro, n mag	kinematc chain, rot mat	KF params, link len	EKF	static, n pose back bent	1, OMC	hand pos	distance to plane 13 circle length 14	-	DH, any kinematic chain
[104]	2013	lower limbs	-	link ori, pelvis pos	method	7 acc, 7 gyro, 7 mag	kinematic chain, quat ori	KF params, link len	KF	-	1, OMC	pelvis pos	pelivs pos *x* 90–120 pelivs pos *y* 40–100 pelivs pos *z* 60–80	-	sens align link, sens sitting link, ZUPT, 20 s hopping valid trial, walking valid trial
[107]	2013	full body		link ori link pos	application	21 acc, 21 gyro, 21 mag	kinematc chain, rot mat	KF params, link len	EKF	static cubic rig poses	MIMUs comparison	-	-	-	no head
[108]	2013	upper limbs	ergonomics	link ori, link pos	application	21 acc, 21 gyro, 21 mag, 2 goniometers	kinematic chain, rot mat	KF params, link len	EKF	static, n pose back bent	12 experts	execution time, RULA class freq	-	-	-
[47]	2013	upper limbs	-	shoulder ori shoulder pos arm ori arm pos forearm ori forearm pos	method	3 acc, 3 gyro, 3 mag	kinematic chain, rot mat	KF params sens pos link len	UKF	static, n pose, t pose	1, OMC	scapula ret scapula ele shoulder abd shoulder rot shoulder fle elbow fle elbow pro shoulder pos elbow pos wrist pos	scapula ret 6.19 scapula ele 3.43 shoulder abd 8.19 shoulder rot 10.68 shoulder fle 8.79 elbow fle 5.00 elbow pro 9.61 shoulder pos 34.1 elbow pos 65.5 wrist pos 103.6	ang 0.63-0.99 pos 0.97-0.99	DH, 160s functional movements valid trials
[65]	2013	lower limbs	localization, training		method	1 acc, 1 gyro, 1 mag, 1 pressure	kinematic chain, rot mat	KF params, link len, sens pos	KF	static, three walk step poses	1, OMC, known path	pelvis vel pelvis pos	pelvis vel 0.03–0.13 pelvis pos 3.2–1832	-	sens sitting link, >40s walking valid trial, >40s jogging valid trial, lin acc noise
[42]	2013	full body	medical	link ori	method	1 acc, 1 gyro, 1 mag	quat ori	PF params	PF, VMF dist	static	1, OMC, robot EE	quat ori quat thigh quat shank	quat ori 0.45-0.6 quat thigh 0.416–0.598 quat shank 0.431-0.606	-	bias est, sens sitting links, init GN opt acc mag meas
[60]	2013	attitude	-	link ori	method	1 acc, 1 gyro, 1 mag	quat ori	CF gain, opt params	CF, GN opt	static, dymanic	1, OMC	roll pitch yaw	roll 0.66 pitch 0.60 yaw 0.82	-	adaptive gain CF, bias from calib, bias est, 1000s valid trials, variation mag disturbance static, lin acc negl
[93]	2014	lower limbs	medical: gait	ankle fle knee fle	method	6 acc, 6 gyro	knee hinge	CF gain	CF like	dynamic	1, OMC	ankle fle knee fle	ankle fle 1.62 knee fle 3.3	-	walk 10m straight valid trial
[79]	2014	lower limbs	medical:gait, rehabilitation	knee fle knee pos ankle pos	method	5 acc, 5 gyro	kinematic chain	KF params	EKF	-	5, OMC	knee fle knee pos ankle pos	knee fle 5.03–6.46 knee pos 0.9–10.3 ankle pos 0.3–11.9	-	2 full cycles valid trial
[70]	2014	upper limbs	-	arm ori arm pos forearm ori forearm pos	method	2 acc, 2 gyro, 2 mag	kinematic chain	U transform, link len, sens pos	PGM	static, n pose, t pose	1, OMC	shoulder abd shoulder rot shoulder fle elbow fle elbow pro	shoulder abd 6.78 shoulder rot 6.64 shoulder fle 3.77 elbow fle 7.24 elbow pro 15.49	shoulder abd 0.94 shoulder rot 0.81 shoulder fle 0.98 elbow fle 0.98 elbow pro 0.74	DH, 160s functional movements valid trials
[55]	2014	lower limbs	-	link pos link ori	method	17 acc, 17 gyro, 17 mag	kinematic chain quat ori rot mat free seg	opt steps, link len	OPT	unspecifieds	1, OMC	knee ori	-	-	bias est, lin acc negl, covmat allanvar, no sens to hinge and acc model, no real time, 37s walking valid trial, show plot
[89]	2014	lower limbs	-	pelvis ori thigh ori shank ori foot ori	calibration	7 acc	rot mat	-	TRIAD like	static, n pose, seat pose	10, OMC	hip fle hip abd hip rot knee fle ankle fle ankle abd ankle rot	hip fle 0.2–0.4 hip abd 0.6–0.9 hip rot 0.4–0.8 knee fle 0.3–0.6 ankle fle 0.6–1.3 ankle abd 0.7–1.9 ankle rot 0.3–0.8	hip fle 0.95–0.99 hip abd 0.87–0.96 hip rot 0.90–0.97 knee fle 0.95-0.98 ankle fle 0.84–0.92 ankle abd 0.69–0.92 ankle rot 0.90–0.97	lin acc negl
[109]	2015	full body	medical: physical activity monitoring	activity class activity rate	application	-	kinematic chain, rot mat	KF params, link len	EKF	static, n pose, back bent	-	-	-	-	
[78]	2015	upper limbs	-	arm ori forearm ori hand ori	method	2 acc, 2 gyro, 2 mag	kinematic chain, rot mat	KF params, link len	UKF	static, n pose	1, mech	shoulder rot shoulder fle elbow fle elbow pro wrist fle wrist twi	shoulder rot 3.0–7.8 shoulder fle 0.8–2.5 elbow fle 0.9–2.8 elbow pro 1.1–1.3 wrist fle 1.1–1.8 wrist twi 1.7–2.8	-	DH, sens align link, sens sitting link, bias est, calib remove gyro bias, ZUPT reduce gyro bias, joint limit, mech synch crosscorrelation, 120s funct mov valid trial, 120 s norm tasks valid trial
[113]	2015	full body	sport	arm ori forearm ori hand ori trunk ori arm pos forearm pos hand pos trunk pos	method, application	5 acc, 5 gyro, 5 mag, 5 enc	kinematic chain, rot mat	KF params, link len	UKF	static	1, OMC	shoulder pos elbow pos wrist pos	shoulder pos 78–81 elbow pos 153–158 wrist pos 34–54	shoulder pos 0.40–0.48 elbow pos 0.83–0.91 wrist pos 0.99	40s rowing valid trial
[110]	2016	upper limbs	ergonomics	arm ori arm pos forearm ori forearm pos	application	3 acc, 3 gyro, 3 mag, EMG	kinematic chain	KF params, link len	UKF	static, n pose, t pose	10, manual vs auto	-	-	-	
[90]	2016	upper limbs	computer graphics	arm ori elbow fle	method	1 acc, 1 gyro, 1 mag, mech track elbow fle	quat ori	KF params, link len	UKF	static, t pose	1, XsensMVN	elbow pos wrist pos	-	-	lin acc negl, sens align link, sens sitting link, 5.5 s valid trial, show plot
[86]	2016	-	-	link ori	mag comp	1 acc, 1 gyro, 1 mag	quat ori	KF params	KF	-	1, OMC	quat ori	quat ori6	-	180s walking valid trial, similar to [32]
[51]	2016	upper limbs	-	link ori link pos	method	3 acc, 3 gyro, 3 mag	kinematic chain rot mat quat ori free seg	KF params link len sens pos sens ori opt params	EKF, OPT	static	1, sim sens meas 1, OMC	arm ori forearm ori wrist ori	arm ori qs 1.68–5.31 forearm ori qs 2.58–8.49 wrist ori qs 2.71–4.57 arm ori qi 1.63–4.66 forearm ori qi 2.40–8.40 wrist ori qi 2.52–4.46 arm ori op 1.27–1.88 forearm ori op 2.16–2.23 wrist ori op 2.28–2.33	-	DH, link len est, sens ori est, motion speed
[91]	2016	full body	-	link ori link pos	assessment	12 acc, 12 gyro, 12 mag	-	link len	-	static	1, OMC	hand ori elbow ori shoulder ori neck ori back ori ankle ori knee ori hip ori	hand ori 5.7–14.1 elbow ori 6.2–12.5 shoulder ori 19.7–40.2 neck ori 3.9–12.3 back ori 4.4–5.9 ankle ori 4.3–7.3 knee ori 3.2–4.1 hip ori 4.0–7.5	hand ori 0.80–0.89 elbow ori 0.81–0.96 shoulder ori 0.39–0.86 neck ori 0.84-0.98 back ori 0.70–0.95 ankle ori 0.77–0.89 knee ori 0.75–0.97 hip ori 0.94–0.97	complex vs simple task valid trials, 1920s manual handling valid trial, error due to biomechanics, total err, ISB kinematic model, MVN kinematic model
[85]	2016	attitude	-	link ori	method	1 acc, 1 gyro, 1 mag	quat ori	KF params	EKF	-	4, OMC	roll pitch yaw	roll 1.0–5.0 pitch 1.6–4.1 yaw 6.2–19	-	bias est, lin acc negl, 120 s texting walking valid trial, 120s swinging walking valid trial, 780 s unsupervised walking valid trial
[105]	2016	lower limbs	-	link ori, pelvis pos	method	7 acc, 7 gyro, 3 UWB	kinematic chain, rot mat	KF params, link len	KF	-	1, OMC	yaw ankle fle knee fle hip fle hip abd	ankle fle 1.4–2.1 knee fle 3.5–4.5 hip fle 2.7–3.4 hip abd 2.9–3.7	-	ZUPT, 100s walking valid trial, 100s jumping valid trial, 100s ascending valid trial

**Table A2:** List of abbreviations.

Abbreviation	Full Name	Categories	Description
ori	orientation	Target, Kinematics & Constraints, Measure, RMSE, Correlation	orientation of a rigid body
pos	position	Target, Calibration, Parameters, Validation, Measure, RMSE, Correlation, notes	position of a point of a rigid body
fle	flexion/extension	Target, Measure, RMSE, Correlation, Sensors,	anatomical term of motion
abd	abduction/adduction	Target, Measure, RMSE, Correlation, Sensors,	anatomical term of motion
rot	rotation	Target, Kinematics & Constraints, Measure, Sensor Fusion Technique, Calibration, RMSE, Correlation	rotation related either to rotation about an axis or rotation matrix
pro	pronation/supination	Target, Measure, RMSE, Correlation, notes
ret	retraction/protraction	Target, Measure, RMSE	scapular retraction/protraction
ele	elevation/depression	Target, Measure, RMSE	scapular elevation/depression
mag	magnetometer	Focus, Sensors, Sensor Fusion Technique, Measure, notes	referred to either 3 axis (unless otherwise specified) magnetometer or its signal
comp	compensation	Focus	referred to compensation of magnetic field distortions
acc	accelerometer	Sensors	referred to either 3 axis (unless otherwise specified) accelerometer or its signal
gyro	gyroscope	Sensors, Sensor Fusion technique, notes	3 axis (unless otherwise specified) gyroscope
xd	-	Sensors	*x* axes sensor (e.g., 2d acc means biaxial accelerometer)
mech	mechanical	Sensors, Validation, notes	mechanical is usually referred to either trackers or rigs for validation
biomech	biomechanical	notes	-
track	tracker	Sensors	-
US	ultrasound	Sensors, Validation	ultrasound sensor or motion tracking system based on ultrasound
exp	exponential	Kinematics & Constraints	exponential maps representation
seg	segment	Kinematics & Constraints	referred to free segments representation
CF	Complementary Filter	Parameters, Sensor Fusion Technique, notes	-
len	length	Parameters, Measures, RMSE, notes	length of human limbs or robotic links
KF	Kalman Filter	Parameters, Sensor Fusion Technique	-
EKF	Extended Kalman Filter	Parameters, Sensor Fusion Technique	-
UKF	Unscented Kalman Filter	Parameters, Sensor Fusion Technique	-
PF	Particle Filter	Parameters, Sensor Fusion Technique	-
PGM	probabilistic grpahical models	Sensor Fusion Technique	-
opt/OPT	optimization	Parameters, Sensor Fusion Technique, notes	-
params	parameters	Parameters	-
sens	sensor(s)	Parameters, Validation, notes	Typically referred to position and orientation of the sensor with respect to the link it is attached to. Otherwise referred to simulated measurements of a virtual sensor
U	unscented	Parameters	-
INS	inertial navigation system	Sensor Fusion Technique	navigation system based on signals from accelerometers and gyroscopes aimed at estimating position, velocity and orientation of a rigid body. It is typically referred to navigation of aerial vehicles
GN	Gauss-Newton		referred to Gauss-Newton optimization
QUEST	Quaternion estimator algorithm	Sensor Fusion Technique	-
err	error	Sensor Fusion Technique, Measure, RMSE	typically error is quantitatively defined as difference of a variable with respect to a reference
int	integration	Sensor Fusion Technique	referred to integration of gyroscope’s or accelerometer’s signal
FQA	Factorized Quaternion Algorithm	Sensor Fusion Technique	-
lin acc	linear acceleration	Sensor Fusion Technique, notes	acceleration of a point in space
sto	stochastic	Sensor Fusion Technique	-
NR	Newton-Raphson	Sensor Fusion Technique	referred to Newton-Raphson algorithm used for optimization
MAG	-	Sensors,	Motion tracking system based on magnetic field measurement
VMF dist	Von Mises-Fisher distribution	Sensor Fusion Technique, notes	-
calib	calibration	Calibration, notes	-
traj	trajectory	Calibration, Measure, RMSE, notes	trajectory of a point in space
EMG	electromyography	Sensors	array of surface electromyography sensors
quat	quaternion	Kinematics & Constraints, Measure, RMSE,	-
coord sys	coordinate system	Kinematics & Constraints	-
EE	end effector	Validation	end effector of a robot
hor	horizontal	Validation	-
ver	vertical	Validation	-
OMC	optical motion capture	Validation, RMSE	OMC is referred to tracking of points in space by means of optical motion capture. Tracking of these points is often used to compute orientation of rigid bodies
sim	simulated	Validation	referred to simulated measurements of virtual sensors
GNSS	global satellite navigation system	Validation	provider of ground truth data in outdoor motion capture sessions
meas	measurement	Validation, notes	-
compar	comparison	Measure	-
incli	inclination	Measure, RMSE	deviation with respect to a given direction
sta	static	Measure, RMSE	-
dyn	dynamic	Measure, RMSE	-
RULA	rapid upper limb assessment	Measure	method of ergonomic assessment based on articular motion and forces exerted during an activity
freq	frequency	Measure	number of occurrence of a risk class in RULA evaluation
twi	twist	Measure, RMSE	referred to wrist motion
valid	validation	notes	referred to validation trials carried out for the validation of the method, typically preceded by their duration
negl	neglected	notes	referred to a variable that is neglected in a method, typically linear acceleration
align	aligned	notes	used in the formula sens align link to indicate the assumption that a sensor’s frame is supposed to be aligned to the frame of the body it is attached to
teleop	teleoperation	notes	-
ROM	range of motion	notes	-
est	estimation	notes	-
alg	algorithm	notes	-
const	constant	notes	-
ZARU	zero angular rate update	notes	technique to reduce drift based on detection of steady orientation
HDR	heuristic heading reduction	notes	technique that exploits straight paths to improve localization estimate
reloc	relocation	notes	referred to relocation of sensors
ang	angle or angular	Correlation, notes	-
insp	inspection	notes	referred to visual inspection
DH	Denavit-Hartenberg	notes	standard to define kinematic chains
synch	synchronization	notes	-
ISB	International Society of Biomechanics	notes	used to refer to the standard proposed by ISB to define frames attached to human limbs to define their pose and motion
UWB	ultra wide band	Sensors	-
